# The KRAB-zinc-finger protein ZFP708 mediates epigenetic repression at RMER19B retrotransposons

**DOI:** 10.1242/dev.170266

**Published:** 2019-07-10

**Authors:** Michelle K. Y. Seah, Yaju Wang, Pierre-Alexis Goy, Hui Mun Loh, Wen Jun Peh, Diana H. P. Low, Brenda Y. Han, Esther Wong, Ei Leen Leong, Gernot Wolf, Slim Mzoughi, Heike Wollmann, Todd S. Macfarlan, Ernesto Guccione, Daniel M. Messerschmidt

**Affiliations:** 1 Developmental Epigenetics and Disease Group, IMCB, A*STAR, 138673, Singapore; 2 Methyltransferases in Development and Disease Group, IMCB, A*STAR, 138673, Singapore; 3 Department of Biochemistry, Yong Loo Lin School of Medicine, National University of Singapore, 117596, Singapore; 4 KOre – Knock Out resource, IMB, A*STAR, 138648, Singapore; 5 The Eunice Kennedy Shriver National Institute of Child Health and Human Development, NIH, Bethesda, MD 20892, USA; 6 NGS Unit of DNA Sequencing Facility, IMCB, A*STAR, 138673, Singapore

**Keywords:** KRAB-ZFP, ZFP708, Epigenetics, Inheritance, Reprogramming, DNA-methylation, Development, Trim28/Kap1, Maternal effect

## Abstract

Global epigenetic reprogramming is vital to purge germ cell-specific epigenetic features to establish the totipotent state of the embryo. This process transpires to be carefully regulated and is not an undirected, radical erasure of parental epigenomes. The TRIM28 complex has been shown to be crucial in embryonic epigenetic reprogramming by regionally opposing DNA demethylation to preserve vital parental information to be inherited from germline to soma. Yet the DNA-binding factors guiding this complex to specific targets are largely unknown. Here, we uncover and characterize a novel, maternally expressed, TRIM28-interacting KRAB zinc-finger protein: ZFP708. It recruits the repressive TRIM28 complex to RMER19B retrotransposons to evoke regional heterochromatin formation. ZFP708 binding to these hitherto unknown TRIM28 targets is DNA methylation and H3K9me3 independent. ZFP708 mutant mice are viable and fertile, yet embryos fail to inherit and maintain DNA methylation at ZFP708 target sites. This can result in activation of RMER19B-adjacent genes, while ectopic expression of ZFP708 results in transcriptional repression. Finally, we describe the evolutionary conservation of ZFP708 in mice and rats, which is linked to the conserved presence of the targeted RMER19B retrotransposons in these species.

## Abstract (precis)

**Summary:** Analysis of the function and targets of a maternal KRAB-zinc-finger protein, ZFP708, found to specifically mediate maintenance of DNA methylation at a subset of LTR retrotransposons during embryonic epigenetic reprogramming.

## INTRODUCTION

The inheritance of selective epigenetic information from germline to soma is crucial for organismal development. This epigenetic memory is remarkably maintained during the near-complete erasure of the germ cell epigenomes that establishes the totipotent embryonic state. The mechanisms mediating this resistance to reprogramming are far from understood. The ‘maintenance’ DNA methyltransferase DNMT1 is essential to this process, creating the paradox of DNMT1 requirement for DNA methylation maintenance while simultaneously enabling global demethylation (Cirio et al., 2008; Hirasawa et al., 2008; Kurihara et al., 2008). In the preimplantation embryo, this is achieved by specific targeting of DNMT1 to selected regions. For example, at genomic imprints, which are paradigmatic for epigenetic inheritance, DNMT1 is recruited directly or indirectly through TRIM28, which is in turn directed to specific genomic loci by the DNA-binding protein ZFP57 (Li et al., 2008; Messerschmidt et al., 2012; Quenneville et al., 2011). ZFP57 is a Krueppel-associated box (KRAB) domain zinc-finger protein (ZFP).

KRAB-ZFPs make up the largest subtype of C2H2-type zinc-finger transcription factors with over 300 genes encoded in the mouse genome (Emerson and Thomas, 2009; Kauzlaric et al., 2017). While mediating sequence-specific binding through their zinc-finger domains, their KRAB domain typically recruits TRIM28 and, in extension, heterochromatin-inducing factors such as histone methyltransferase SETDB1, the nucleosome remodeling and histone deacetylase (NuRD) complex, and DNA methyltransferases 3A, 3B and 1 (Ecco et al., 2017; Friedman et al., 1996; Quenneville et al., 2011; Schultz et al., 2002, 2001; Urrutia, 2003). It has been proposed that KRAB-ZFPs and their mode of action have, in the first instance, emerged in an evolutionary struggle to combat and/or tame endogenous retroviral elements (EREs) (Ecco et al., 2017; Jacobs et al., 2014; Thomas and Schneider, 2011; Wolf et al., 2015a). However, several lines of evidence suggest that KRAB-ZFPs may have evolved from mere ERE-repressing guardians of the genome to important developmental regulators, as shown for ZFP57 or ZFP568 (Li et al., 2008; Yang et al., 2017). KRAB-ZFP are indeed ideally positioned to regionally (through their DNA-binding specificity) modulate epigenetic states (through the effectors recruited by TRIM28). This is of particular importance during preimplantation development, where epigenetic marks, such as genomic imprints protected by ZFP57, are maintained despite global epigenetic reprogramming (Li et al., 2008; Messerschmidt et al., 2012; Quenneville et al., 2011). Here, TRIM28 function is crucial and its maternal deletion alone not only causes imprinting defects but a large range of developmental defects, including male-specific early embryonic lethality (Lorthongpanich et al., 2012; Messerschmidt et al., 2012; Sampath Kumar et al., 2017). Apart from the ZFP57-mediated imprint maintenance, it is not known how and to which regions maternal TRIM28 is targeted, but maternally expressed KRAB-ZFP proteins are likely candidates. Yet to date, apart from ZFP57, no other maternal KRAB-ZFP has been investigated in the context of embryonic epigenetic reprogramming. Here, we identify and study a previously uncharacterized KRAB-ZFP, ZFP708, which, based on its maternal expression, holds great promise for mediating oocyte-to-embryo-specific functions of the KRAB-ZFP/TRIM28 complex. Indeed, we show here that ZFP708 is needed to carry over DNA methylation at RMER19B LTR-retrotransposons from germline to soma, expanding our understanding of the KRAB-ZFP/TRIM28-protected heritable epigenome.

## RESULTS

### ZFP708 is a maternal TRIM28-interacting KRAB-zinc-finger protein

**Fig. 1.**
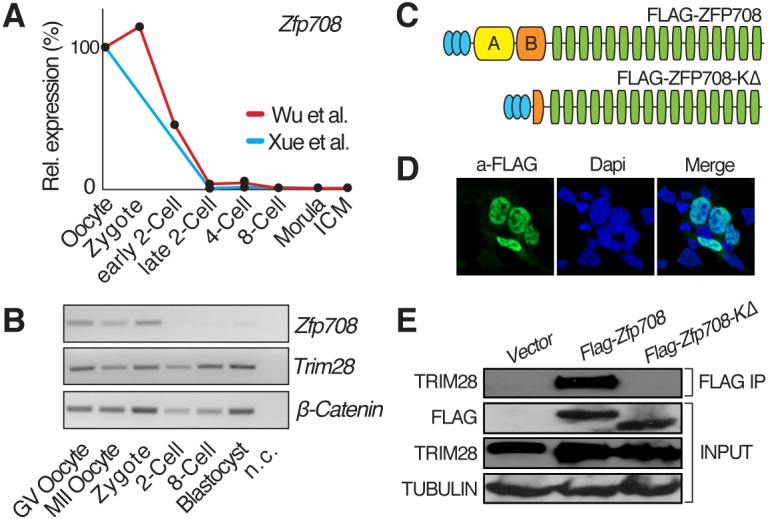
**ZFP708 is a maternal TRIM28-interacting KRAB-ZFP.** (A) ZFP708 expression across oocytes and preimplantation stages derived from two independent published RNA-seq data sets. RPKM values were normalized to β-actin and are shown relative to oocyte expression levels. (B) Semi-quantitative RT-PCR expression analysis across oocytes and embryos for *Zfp708*, *Trim28* and β-catenin (loading control) [GV, germinal vesicle; n.c., negative (water) control]. (C) Schematic illustration of cloned, FLAG-tagged ZFP708 variants (top, full-length ZFP708; bottom, KRAB-domain deleted ZFP708) (blue ovals, FLAG epitopes; yellow box, KRAB A-domain; orange box, KRAB B-domain or fragment thereof; green boxes, zinc fingers). (D) Immunofluorescence staining for FLAG-tagged ZFP708 overlapping with nuclear DAPI staining. (E) Co-immunoprecipitation of FLAG-tagged full-length and FLAG-tagged KRAB-deleted ZFP708 variants with TRIM28. As negative control, empty expression vector was transfected (Vector).

To shed light into the functions and targets of maternal TRIM28 during oocyte to embryo transition and early preimplantation development, we explored the expression patterns of potential TRIM28-interacting KRAB-ZFP transcription factors in mouse oocytes and at early embryonic stages. We identified *Zfp708* (also known as *Rslcan11*) as a candidate of interest in two independently published RNA-sequencing datasets exploring oocyte and preimplantation embryo transcriptomes (Wu et al., 2016; Xue et al., 2013). *Zfp708* is highly expressed in oocytes and zygotes, but RNA levels drastically decrease at the two-cell stage and remain low or undetectable in subsequent preimplantation stages, including the ICM of the blastocyst (Fig. 1A). We confirmed these observations using semi-quantitative RT-PCR (Fig. 1B). As opposed to the oocyte, where *Zfp708* is among the KRAB-ZFPs with highest relative expression, mouse ENCODE transcription data (Table S1) and other public datasets show low to no expression levels at later stages or adult tissues.

*Zfp708* is located on mouse chromosome 13 and is part of a typical KRAB-ZFP cluster containing at least 23 other family members, first described as *Rslcan* (regulator of sex-limitation candidate) genes (Krebs et al., 2009, 2005). It encodes a classical, well-conserved N-terminal KRAB domain consisting of a KRAB-A and a KRAB-B box followed by 16 predicted C2H2 zinc fingers (Fig. 1C and Fig. S1). The presence of intact C2H2 zinc-finger domains suggests DNA-binding potential; the KRAB-A box is a known docking platform for TRIM28.

We cloned the full-length *Zfp708* transcript corresponding to the annotated isoform A (NM_001012325.2) from ovary cDNA. We failed in several attempts to derive specific antibodies for ZFP708. This is a frequent problem owing to the repetitive nature of the zinc-finger arrays and high KRAB domain conservation in KRAB-ZFPs. We therefore introduced an N-terminal (3x) FLAG-tag for downstream analyses (Fig. S1A). Immunofluorescence staining of FLAG-ZFP708 in mouse embryonic stem cells (mESCs) revealed robust expression and clear nuclear localization (Fig. 1D). Co-immunoprecipitation of FLAG-ZFP708 with endogenous TRIM28 in FLAG-ZFP708-overexpressing (OE) mESCs confirmed the expected physical interaction between the two proteins. Predictably, this interaction is KRAB domain dependent, as removal of the whole KRAB-A and partial KRAB-B box from ZFP708 (FLAG-ZFP708-KΔ) (Fig. 1C and Fig. S1A and B) disrupts TRIM28 interaction (Fig. 1E). ZFP708 is therefore a *bona fide* maternal, TRIM28-interacting, KRAB-zinc-finger protein.

### Identification of ZFP708-binding sites

**Fig. 2.**
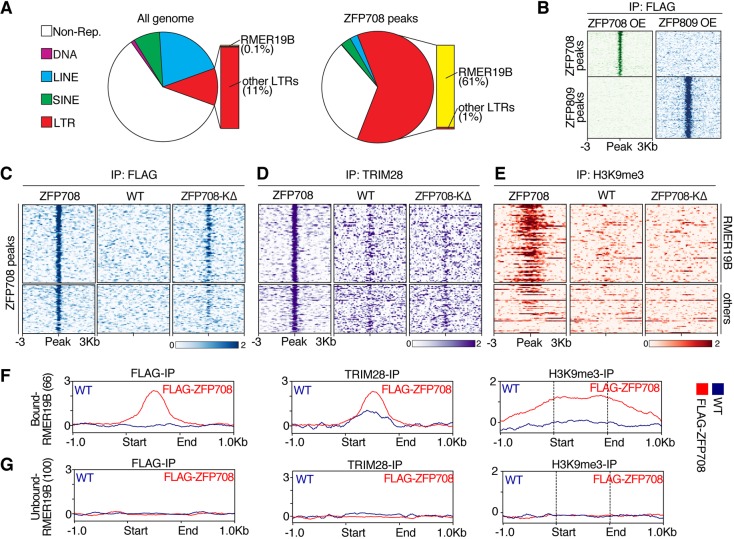
**ZFP708 binds to RMER19B elements of the ERVK family of LTR retrotransposons.** (A) Distribution of genomic features across whole-genome and FLAG-ZFP708 peaks. (B) Comparison of peaks identified by Flag-ChIP using FLAG-tagged ZFP708- or FLAG-tagged ZFP809-expressing (Wolf et al., 2015b) mESCs. (C-E) Heat maps of FLAG (C), TRIM28 (D) and H3K9me3 (E) enrichment at ZFP708 ChIP-seq peak regions in ZFP708-overexpressing mESCs (left), wild-type mESCs (middle) and ZFP708-KΔ-overexpressing mESCs (right). Peak summits are centered and 3 kb regions up- and downstream are displayed. (F,G) FLAG, TRIM28 and H3K9me3 enrichment at ZFP708-bound (F, *n*=66) and least-bound (G, *n*=100) RMER19B elements. EREs were fitted to 1 kb in length; additional 1 kb regions up- and downstream are displayed.

TRIM28 complex targeting through KRAB-ZFPs to specific sites crafts regional epigenetic changes or, during embryonic reprogramming, can preserve regional DNA methylation (Messerschmidt et al., 2014). We sought to identify ZFP708 target sites by ectopically expressing FLAG-ZFP708 in mESCs for chromatin immunoprecipitation followed by deep sequencing (ChIP-seq) (Fig. 2 and Figs S2-S4). The identified, high-confidence binding sites across three independent experiments showed a notable enrichment over long terminal repeat (LTR) EREs compared with the genome-wide distribution of these elements (Fig. 2A). More strikingly still, 93% of these LTR-underlying ZFP708 peaks (and more than half overall) were identified as ERVK family RMER19B elements (Fig. 2A) (Tables S2-S5). To validate our FLAG-tag KRAB-ZFP ChIP-seq approach, we compared the FLAG-ZFP708 targets with those of a published but unrelated FLAG-tagged KRAB-ZFP: ZFP809 (Wolf et al., 2015b). Although both transcription factors specifically bind to LTR retrotransposons, their actual binding sites and targeted ERE subfamilies do not overlap, arguing for the specificity of each factor and validity of the approach (Fig. 2B). In addition, we found similar binding specificity and substantial overlap of binding sites in an independent ChIP-seq approach using an HA-tagged version of ZFP708 (Fig. S2A-D).

FLAG ChIP-seq on control (wild-type) mESCs showed no enrichment at the newly identified FLAG-ZFP708-binding sites (Fig. 2C). Instead, the KRAB domain-deleted ZFP708 variant (ZFP708-KΔ) still showed binding to these sites, albeit with reduced efficiency, indicating a zinc finger-dependent interaction of ZFP708 with RMER19B elements (Fig. 2C).

### Recruitment of TRIM28 and H3K9me3 to RMER19B elements by ZFP708

Detailed studies showing TRIM28 binding to and regulation of an extensive array of EREs in mESCs have not described RMER19B elements as TRIM28 targets to date (Castro-Diaz et al., 2014; Rowe et al., 2010). We re-examined five independently published mESC TRIM28 ChIP-seq datasets (Castro-Diaz et al., 2014; Quenneville et al., 2011; Riso et al., 2016; Rowe et al., 2010) and found only 0.3-0.8% of all peaks mapping to LTR retrotransposons belonging to the RMER19B family (Fig. S2E). We observed similar TRIM28 peak overlap frequency to RMER19B elements in our own ChIP-seq datasets from control or ZFP708-KΔ-overexpressing mESCs (0.4 and 0.3%, respectively) (Fig. S2E). In mESCs overexpressing intact ZFP708, however, the RMER19B overlap is increased to 2.5% of all LTR-overlapping TRIM28 peaks (Fig. S2E). Ectopically expressed ZFP708 allows for efficient recruitment of TRIM28 to all FLAG-ZFP708-bound sites, whereas peak overlap with TRIM28 in control and ZFP708-KΔ-overexpressing cells is minimal (Fig. 2D and Fig. S2F). Similarly, minimal overlap is found with the aforementioned five published TRIM28 ChIP-seq datasets (Fig. S2G,H), which is in line with the marginal expression of endogenous ZFP708 in mESCs. The recruitment of TRIM28 to ZFP708 sites in the ZFP708 overexpression ESCs correlates with a specific increase of H3K9me3, suggesting the recruitment of not just TRIM28 but the whole KRAB-ZFP/TRIM28 machinery (Fig. 2E). As for TRIM28 enrichment, no H3K9me3 accumulation was found at ZFP708-binding sites in our own control (non-ZFP708-expressing wild type) mESCs (Fig. 2E) or published datasets for SETDB1 and H3K9me3 ChIP in wild-type mESCs (Fig. S2H) (Dan et al., 2014; Yuan et al., 2009). Finally, although the KRAB domain-deleted ZFP708 variant still binds to its target regions, TRIM28 recruitment and H3K9me3 accumulation to ZFP708-binding sites in FLAG-ZFP708-KΔ-overexpressing mESCs is abolished (Fig. 2C-E).

Our stringent ZFP708-binding analysis, achieved by overlapping three replicate ChIP-seq datasets, may result in underestimation of the number of RMER19B elements bound. Indeed, enrichment analysis of the individual data supports this notion (Fig. S3A). Comparing the RMER19B elements bound in all three experiments (66) with the 100 RMER19B elements showing least enrichment we can, however, confirm the clear correlation of TRIM28 and H3K9me3 recruitment to bound elements versus unbound elements (Fig. 2F,G). Notably, H3K9me3 extends into the bound RMER19B element adjacent regions, as shown for numerous heterochromatic regions in mammalian genomes (Fig. 2F). As observed in the initial peak analysis, ZFP708 enrichment is specific to RMER19B and no substantial enrichment is found at closely related RMER19A, RMER19C and other, unrelated, retrotransposons (Fig. S3A-D).

### DNA methylation- and H3K9me3-independent binding of ZFP708 to RMER19B

As shown for ZFP57, KRAB-ZFP binding to target sites can be DNA methylation dependent (Liu et al., 2012; Messerschmidt et al., 2012; Quenneville et al., 2011). To test whether ZFP708 shows similar properties, we overexpressed FLAG-ZFP708 in mESCs lacking DNA methylation by virtue of a triple knockout of *Dnmt1*, *Dnmt3a* and *Dnmt3b* (DNMT TKO). ChIP-qPCR analysis of three prominent targeted RMER19B elements show that ZFP708 binding to DNA is evidently DNA methylation independent (Fig. S4B-D). Our findings further suggest that DNA binding of ZFP708 is independent of pre-existing H3K9me3 or TRIM28/SETDB1, which are absent at identified ZFP708 target sites in mESCs prior to ZFP708 overexpression (Fig. 2C,D and Fig. S2).

Thus, by ectopically expressing ZFP708 in mESCs, we were able to identify RMER19B elements as its likely targets in early embryos. Moreover, we were able to show the KRAB domain-dependent, functional recruitment of TRIM28 and H3K9me3 to these sites. ZFP708 binding is highly specific and independent of pre-existing H3K9me3 and DNA methylation.

### ZFP708 binding to RMER19B elements results in transcriptional repression

**Fig. 3.**
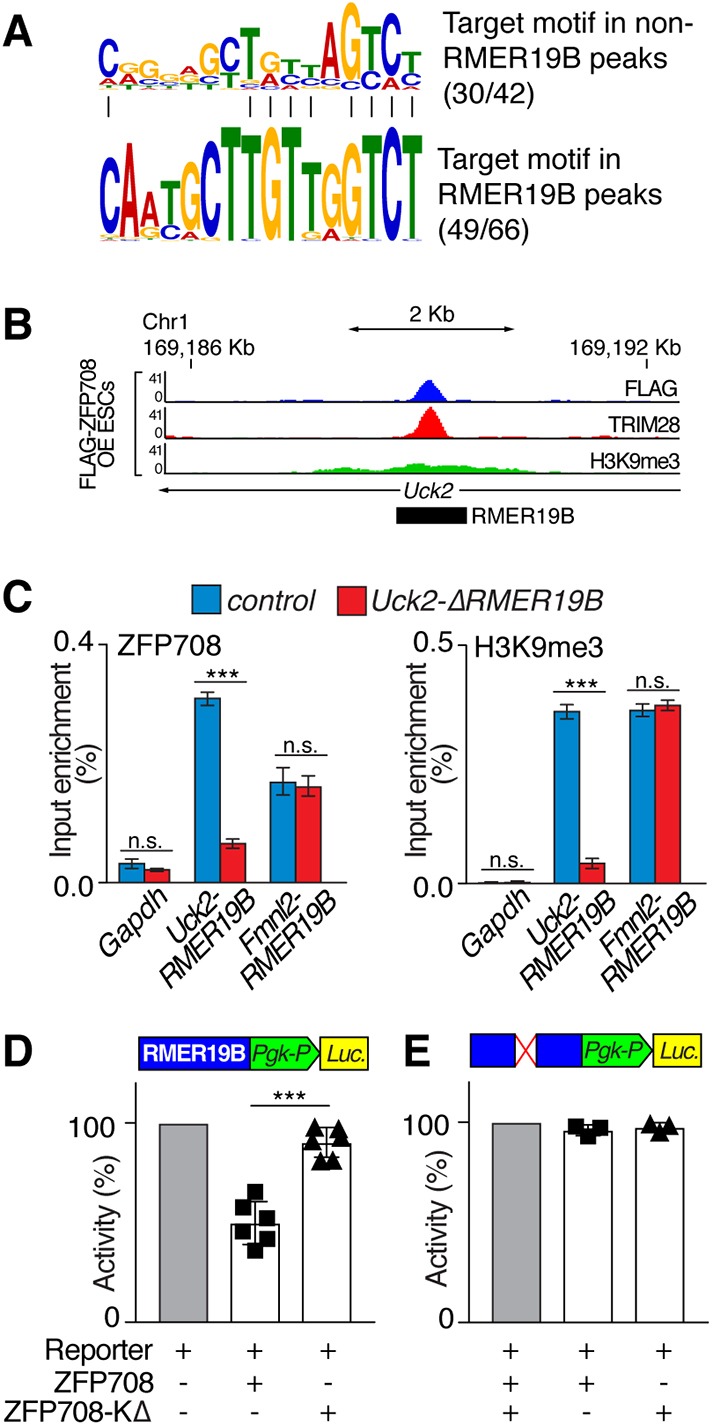
**ZFP708 binding to and effects on RMER19B elements.** (A) Predicted binding motifs using non-RMER19B and RMER19B peaks. (B) Genome browser view of ZFP708, TRIM28 and H3K9me3 enrichment in FLAG-ZFP708 OE ESCs at the *Uck2-RMER19B* locus. (C) ChIP-qPCR analysis of ZFP708 and H3K9me3 enrichment at two RMER19B elements in wild-type control (blue) and *Uck2-ΔRMER19B* (red) mESCs (*n*=3 technical replicates; one representative of *n*=3 independent experiments is shown). (D) Repressive effect of ZFP708 or ZFP708-KΔ on *Uck2-RMER19B* element in luciferase assay. Relative activity (%) compared with vector-only transfected controls of *n*=6 independent experiments is shown (48 h post-transfection). (E) Same experiment as shown in D using the ZFP708-binding site-deleted *Uck2-ΔRMER19B* element. Data are mean±s.d. of at least *n*=3 independent experiments. *P*-values were determined using an unpaired, two-tailed *t*-test: ***P<0.0005.

We used non-RMER19B-overlapping and RMER19B-overlapping peaks to independently predict a putative consensus-binding motif for ZFP708 (Tables S4, S5). The best prediction, found in 71% and 74% of sequences, respectively, revealed a strong motif, which was furthermore similar between both peak sets (Fig. 3A and Fig. S5A). Among the closely related RMER19A and RMER19C elements, this motif is specific to RMER19B (Fig. S5), presumably resulting in the exclusive targeting of these elements (Fig. S6). Using this predicted motif, we addressed ZFP708 binding in a locus-specific manner. To this end, we focused on an intronic RMER19B insertion at the *Uck2* gene locus on chromosome 1 (henceforth referred to as *Uck2-RMER19B*), for which ChIP-seq analysis revealed strong binding for ZFP708- and ZFP708-dependent TRIM28/H3K9me3 recruitment/enrichment (Fig. 3B and Fig. S5C). Using CRISPR/CAS9, we targeted the RMER19B-binding motif found at this site and identified a mESC clone carrying a homozygous 77 bp deletion, including the predicted binding site, hereafter named *Uck2-ΔRMER19B* (Fig. S5D,E). ChIP-qPCR confirmed strong enrichment of ZFP708 at the wild-type *Uck2-RMER19B* locus in FLAG-ZFP708 OE mESCs, as observed in our ChIP-seq findings. However, in *Uck2-ΔRMER19B*/FLAG-ZFP708 OE mESCs, this binding was significantly reduced (Fig. 3C and Fig. S5B). In addition, H3K9me3 enrichment was reduced significantly at the *Uck2-ΔRMER19B* locus compared with the non-targeted *Uck2-RMER19B* site (Fig. 3C). On the other hand, ZFP708 and H3K9me3 remained enriched and unchanged over another ZFP708-targeted, yet not mutated, RMER19B element (*Fmnl2-RMER19B*) (Fig. 3C).

KRAB-ZFPs have been shown to act as repressors of ERE activity through the recruitment of TRIM28, SETDB1 and other complex components (Ecco et al., 2016; Wolf et al., 2015b). We cloned the full-length *Uck2-RMER19B* retrotransposon into a PGK promoter-driven luciferase construct to test the repressive activity of ZFP708 on this element. Co-transfection of the reporter with *Flag-Zfp708* resulted in significant reduction of luciferase activity, suggesting the ZFP708-mediated recruitment of the repressive TRIM28 machinery to the RMER19B element (Fig. 3D). Conversely, the co-transfection of the TRIM28-interaction incompetent *Flag-Zfp708-KΔ* construct did not result in relevant repression (Fig. 3D). Furthermore, neither FLAG-ZFP708 nor FLAG-ZFP708-KΔ had any effects on the CRISPR-induced ZFP708-binding site-deleted *Uck2-ΔRMER19B* in the luciferase assay (Fig. 3E).

### Epigenetic dynamics at RMER19B elements in early embryos

**Fig. 4.**
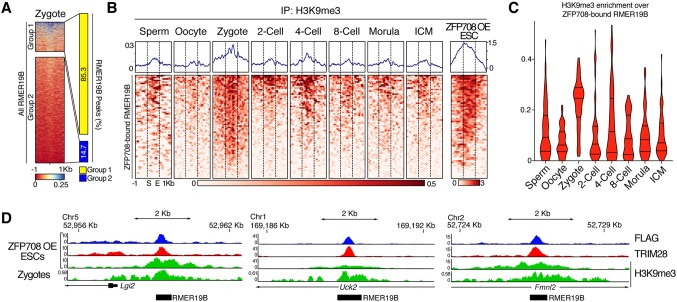
**H3K9me3 dynamics at ZFP708-bound RMER19B elements in preimplantation embryos.** (A) K-means clustering of H3K9me3 enrichment at RMER19B elements in mouse zygotes and ZFP708-bound RMER19B element distribution among resulting clusters. (B) Heat maps of H3K9me3 enrichment dynamics at ZFP708-bound RMER19B elements in sperm, oocytes and preimplantation embryos (Wang et al., 2018). EREs were fitted to 1 kb in length; additional 1 kb regions up- and downstream are displayed. (C) Violin plots quantifying the H3K9me3 enrichment across the ZFP708-bound RMER19B elements displayed in B, omitting the flanking regions. (D) Exemplary genome browser views of ZFP708-bound RMER19B insertions at the *Lgi2*, *Uck2* and *Fmnl2* gene loci showing FLAG-ZFP708, TRIM28 and H3K9me3 enrichment in ZFP708-overexpressing mESCs and H3K9me3 enrichment in wild-type zygotes (Wang et al., 2018).

Global H3K9me3 dynamics have recently been described in preimplantation mouse embryos using ultra-low input ChIP-seq methods (Wang et al., 2018). This work shows that maternal and paternal H3K9me3 marks are extensively reprogrammed shortly after fertilization and a gain, particularly in LTR retrotransposons, is observable in the newly formed embryo. We mined the available data specifically for H3K9me3 dynamics at ZFP708-bound and -unbound RMER19B elements in germ cells and early embryos (Fig. 4 and Fig. S7). We first performed unsupervised K-means clustering of H3K9me3 domains at all RMER19B elements in zygotes into two groups: group 1 with high levels of H3K9me3; group 2 with low levels of H3K9me3. Out of the almost 6000 RMER19B insertions annotated in the mouse genome (mm9), only a small fraction showed H3K9me3 enrichment in zygotes (Fig. 4A). Notably, 85% of the ZFP708-bound RMER19B elements identified in our ZFP708 OE mESCs overlap with group 1 regions, suggesting a correlation of ZFP708 binding and H3K9me3 enrichment *in vivo*. We next focused on the ZFP708-bound RMER19B regions in more detail (Fig. 4B-D). No apparent H3K9me3 enrichment was found at ZFP708-bound RMER19B regions (where histones are detectable) in sperm. Similarly, in oocytes the H3K9me3 density is low at ZFP708-bound RMER19B elements. In zygotes, however, a prominent increase of H3K9me3 is found at these regions (Fig. 4B-D). This is in line with a general post-fertilization increase of H3K9me3 at LTR retrotransposons in the embryonic genome (Wang et al., 2018). Yet, in contrast to the majority of LTR elements that display a continuous increase of H3K9me3 throughout preimplantation development (Wang et al., 2018), the H3K9me3 levels on ZFP708-bound RMER19B elements peaked in the zygote and were decreased at subsequent stages (Fig. 4B,C and Fig. S7A-C). Remarkably, ZFP708-unbound RMER19B elements and ZFP708-bound non-RMER19B regions do not recapitulate these dynamics and show little to no H3K9me3 enrichment and changes throughout preimplantation development (Fig. S7D-G). In conclusion, ZFP708-bound RMER19B elements identified with our overexpression approach in mESCs gain H3K9me3 transiently post-fertilization, a dynamic correlation with the ZFP708 expression in the preimplantation embryo.

### ZFP708 knockout mice are viable and fertile

**Fig. 5.**
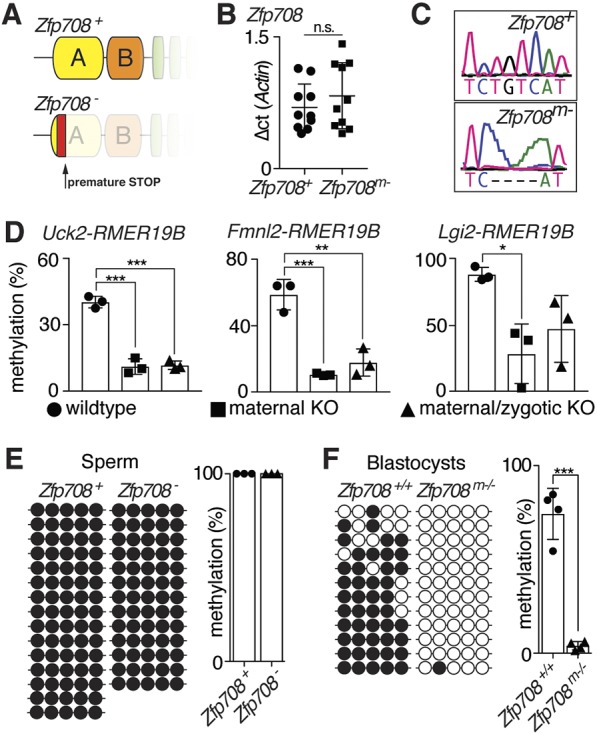
**ZFP708 maintains DNA methylation.** (A) Scheme of wild-type and predicted mutant ZFP708 protein. (B) qRT-PCR for *Zfp708* in wild-type and mutant GV oocytes. ΔCt for 10 embryos are plotted. (C) cDNA sequencing of CRISPR-targeted region shows four bp deletion in the mutant *Zfp708* allele. (D) DNA methylation levels at three ZFP708-bound RMER19B elements in mESCs derived from wild-type, maternal and maternal/zygotic *Zfp708* knockout blastocysts. Average DNA methylation (%) for *n*=3 independently isolated mESC lines for each genotype is shown. (E) DNA methylation state at *Uck2-*RMER19B in wild-type and mutant sperm. Shown is a representative wild-type and mutant sample, and the average DNA methylation (%) across *n*=3 biological replicates for each genotype (see Fig. S12). (F) DNA-methylation state of *Uck2-RMER19B* in wild-type and maternal mutant *Zfp708* blastocysts. Shown is a representative sample and the average DNA methylation (%) across *n*=4 samples for each genotype. Each circle represents a CpG site; white circle, unmethylated; black circle, methylated. Data are mean±s.d. *P*-values were determined using unpaired, two-tailed *t*-test are as follows: ***P<0.0005, **0.0005<*P*<0.005, *0.005<*P*<0.05.

We established *Zfp708* knockout mice using the CRISPR/CAS9 method targeting the KRAB-domain region to address ZFP708 function *in vivo*. We identified a heterozygous founder line with a four-nucleotide deletion producing a frame-shift mutation followed by an early translation termination site. The predicted resulting truncated protein lacks both KRAB and zinc-finger domains (Fig. 5A and Fig. S8A). Although qRT-PCR in mutant and control oocytes did not show nonsense-mediated decay (NMD) of *Zfp708* mRNA, cloning and sequencing of RT-PCR products confirmed the deletion of four nucleotides at the CRISPR target site as identified at the genomic level (Fig. 5B,C). Intercross of heterozygous *Zfp708^+/−^* mice gave rise to viable pups in expected mendelian ratios. The identified homozygous (*Zfp708*^−/−^) animals showed no overt physical abnormalities and displayed normal feeding and breeding behavior compared with control mice in our facility. Both male and female *Zfp708*^−/−^ animals are fertile.

### *Zfp708* knockout results in loss of DNA methylation *in vitro* and *in vivo*

It has been shown that KRAB-ZFP/TRIM28-mediated initial silencing through H3K9me3 in early embryos is epigenetically heritable through DNA methylation (Quenneville et al., 2012; Rowe et al., 2013; Wiznerowicz et al., 2007). ZFP57, TRIM28 and DNMT1, on the other hand, directly maintain DNA methylation of imprints in early embryonic stages (Lorthongpanich et al., 2013; Messerschmidt et al., 2012; Quenneville et al., 2011). Despite the lack of an evident phenotype in the *Zfp708* knockout, we turned to investigate the molecular, epigenetic consequences of *Zfp708* loss during early embryonic development. ZFP708 is not expressed in wild-type mESCs and ZFP708-targeted RMER19B elements are generally not H3K9me3 enriched (Fig. 2C-E). To address the impact of ZFP708 on DNA methylation, we chose three ZFP708/TRIM28-targeted RMER19B elements: *Uck2-RMER19B*, *Fmnl2-RMER19B* and *Lgi2-RMER19B* (Figs S9-S11). We then addressed their methylation state in mESC lines derived from control (*Zfp708^+/+^*), maternal-null (*Zfp708^mat−/+^*) and maternal/zygotic-null (*Zfp708^mat−/−^*) blastocysts under 2i conditions (see Fig. S8C for a detailed mating scheme). Despite the prevalent global hypomethylated state under 2i conditions, the examined ZFP708-targeted RMER19B sites retained notable levels of DNA methylation with 44±6.9%, 53.6±3.5% and 88±5.2%, respectively, at the three tested loci in control lines (Fig. 5D and Figs S9-S11). The *Oct4* promoter was found to be fully hypomethylated in all cell lines (Fig. S12A). Absence of maternal as well as maternal/zygotic ZFP708 in the embryos from which the mESCs were derived, consistently resulted in substantial hypomethylation across all three examined loci (14.8±4.6%/15.6±2.5%, 7.9±0.5%/13.3±6.11% and 28±22.44%/46.9±25.1% at *Uck2-RMER19B*, *Fmnl2-RMER19B* and *Lgi2-RMER19B*, respectively) (Fig. 5D and Figs S9-S11). In wild-type sperm, the *Uck2-RMER19B* locus is fully methylated and mutant-derived sperm show equally high methylation levels (Fig. 5E and Fig. S12C). In contrast to the global DNA demethylation in preimplantation embryos, elevated DNA methylation levels persist and are also found in control blastocysts (74.3±13.6%). Notably, however, lack of maternal ZFP708 results in a near-complete loss of DNA methylation at the *Uck2-RMER19B* in blastocysts (Fig. 5F and Fig. S12D). Hence, maternal ZFP708 is required for the maintenance of DNA methylation marks at its target sites in preimplantation embryos.

### The transcriptional impact of ZFP708 on RMER19B elements and neighboring genes

**Fig. 6.**
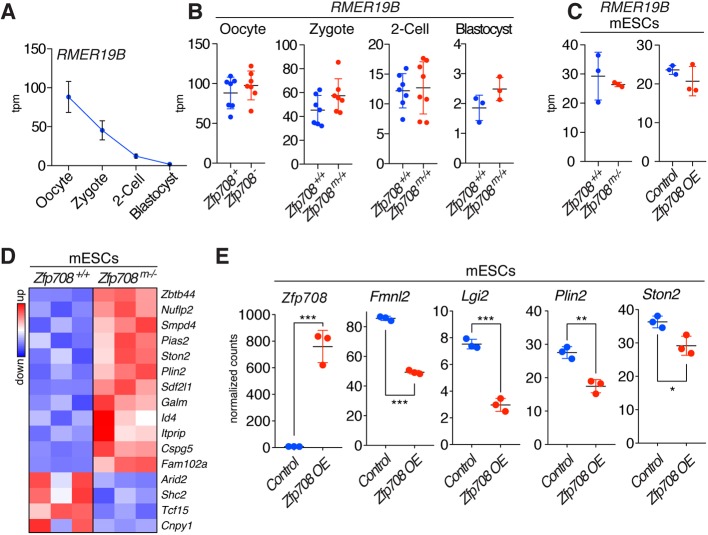
**ZFP708-dependent transcriptional effects in embryos and mESCs.** (A) RMER19B expression in oocytes, zygotes, two-cell embryos and blastocysts. (B) Differential expression of RMER19B in control and maternal knockout oocytes and embryos (zygotes, two-cell stage embryos and blastocysts). (C) RMER19B expression in maternal/zygotic blastocyst-derived and wild-type mESCs (left panel) and in FLAG-ZFP708 OE versus control mESCs (right panel). Data are mean±s.d.; expression changes are not significant (*P*>0.05; unpaired, two-tailed *t*-test). (D) Gene expression analysis in maternal/zygotic blastocyst-derived and wild-type mESCs. Significantly differentially expressed genes within 50 kb of the ZFP708-bound RMER19B elements are shown in a heat map (row z-score). (E) Selected differentially expressed genes in ZFP708 OE mESCs. *Zfp708* is highly overexpressed, ZFP708-bound RMER19B adjacent genes are significantly downregulated. Data are mean±s.d.; *P*-values were calculated using an unpaired, two-tailed *t*-test and are as follows: ***P<0.0005, **0.0005<*P*<0.005, *0.005<*P*<0.05. tpm, transcripts per million mapped reads.

Loss of KRAB-ZFP/TRIM28-mediated repression can result in direct or indirect transcriptional activation of EREs (IAPs, VL30, etc.) and/or neighboring genes, respectively (Rowe et al., 2010; Wolf et al., 2015b). To assess potential transcriptional changes in RMER19B elements, we performed RNA-sequencing on GV oocytes, zygotes, two-cell stage embryos, blastocysts and mESCs derived from maternal/zygotic mutant blastocysts and mESCs overexpressing ZFP708 (Fig. 6).

In normal development, RMER19B expression is highest in GV oocytes and successively decreases after fertilization to very low levels at the blastocyst stage (Fig. 6A). Yet, overall expression levels are low in comparison with well-studied EREs found in oocytes and preimplantation stage embryos (e.g. MTAs or MERVLs) (Fig. S13A,B, Table S6) (Macfarlan et al., 2012; Peaston et al., 2004). The lack of maternal ZFP708 does not result in significant RMER19B expression changes in oocytes and embryos or in mESCs derived from mutant blastocysts (Fig. 6B,C). Finally, overexpression of ZFP708 in mESCs did not affect RMER19B expression levels either (Fig. 6C).

We next examined potential collateral expression effects on genes neighboring ZFP708-bound RMER19B elements (genes with transcription start sites up- or downstream within 50 kb of ChIP-seq peak; Table S7). In mESCs derived from maternal/zygotic mutant blastocysts, we found that of 16 genes showing significant expression changes, 12 are upregulated, as expected (Fig. 6D, Table S8). Conversely, we found that overexpression of ZFP708 in mESCs can negatively impact on ZFP708-bound RMER19B adjacent genes (Fig. 6E, Table S9). *In vivo*, these transcriptional changes were less pronounced and variable across individual embryos (Tables S10-S13). No significant deregulation was detected among the genes in blastocysts and GV oocytes. In zygotes, of five significantly regulated peak-adjacent genes, two showed mildly increased expression (including *Uck2*; Fig. S13C). Although at the two-cell stage we do observe a number of differentially expressed genes adjacent to the analyzed peak regions, these embryos show overall highest variability in gene expression, possibly reflecting stage differences in relation to zygotic gene activation (Fig. S13D).

In summary, the transcriptional effects caused by the maternal loss of ZFP708 are, if at all detectable, mild and variable in preimplantation embryos. RMER19B expression itself is not affected and is generally low in embryos and mESCs. Gene misregulation, possibly through neighboring effects, is prominent in mutant-derived mESCs and possibly reflects the loss of epigenetic repressive marks in absence of ZFP708, in line with our observation of lost DNA methylation at ZFP708-bound RMER19B elements (Fig. 5).

### Evolutionary conservation of ZFP708

**Fig. 7.**
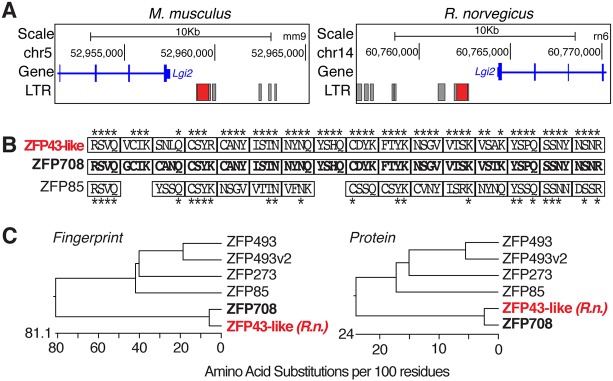
**Evolutionary conservation of RMER19B insertions and ZFP708 in mouse and rat.** (A) RMER19B insertions are highly conserved in mice and rats. Genome browser views of the 5′ region of the *Lgi2* gene, including LTRs. The ZFP708-bound RMER19B in the mouse *Lgi2* promoter (left) and its syntenic insertion in rat (right) are highlighted in red. (B) ‘Fingerprint’ comparison of closely related rat ortholog ZFP43-like and closest mouse paralog ZFP85. Amino acid residues immediately preceding the alpha-helix (position −1) and the second, third and sixth amino acid within the helix were retrieved as the fingerprint for each zinc finger. Asterisks label conserved residues between ZFP708 and rat ZFP43-like and mouse ZFP85. (C) Phylogenetic analysis of proteins closely related to ZFP708 at the ‘fingerprint’ (left) or overall sequence (right) levels. Rat ZFP43-like is labeled in red.

The importance of a molecular mechanism, despite the lack of overt phenotype and mild transcriptional effects if disturbed, may be reflected in its conservation throughout evolution. RMER19B elements are conserved in *Muridae* and readily detectable in the rat genome, yet not in more distant rodents such as the Chinese hamster. Indeed, comparable with the mouse genome, ∼6000 RMER19B insertions are found in rat and ZFP708-bound RMER19B insertions in mouse, such as the *Uck2-RMER19B*, *Fmnl2-RMER19B* and *Lgi2-RMER19B*, are well-conserved in their syntenic regions in the rat genome (Fig. 7A). Likewise, a BLAST search for ZFP708 orthologs uncovered one particularly conserved protein encoded on rat chromosome 2q22 with over 90% amino acid identity (Fig. S14). Chr2q22 was previously described as one of two syntenic regions for the mouse *Rsl* locus in rat, yet the putative ZFP708 ortholog was not identified in that study (Krebs et al., 2005). Despite close conservation at the amino acid level, closely related KRAB-ZFPs may still diverge on binding properties. Defined by four DNA-interacting residues within the zinc fingers (Liu et al., 2014), the specific ‘fingerprint’ is, however, equally conserved between the mouse and putative rat ortholog (Fig. 7B). The conservation of mouse ZFP708 and its rat homolog is indeed higher than the conservation with any other mouse KRAB-ZFP paralog we could identify, reflected in the clustering comparing overall protein identity or conservation of the ‘fingerprints’ alone (Fig. 7C). The closest conserved KRAB-ZFP in humans still lies within the syntenic *Rsl* locus on HSA19p12-13, yet conservation is far lower, consistent with the absence of RMER19B elements in the human genome. It therefore appears that in *Muridae* ZFP708 has evolved within the *Rsl* locus specifically in response to the invasion and threat of the RMER19B EREs.

## DISCUSSION

In conclusion, we have identified a novel, maternal, TRIM28-interacting KRAB-ZFP (ZFP708) that specifically targets LTR class endogenous retroviral elements of the ERVK family: RMER19B. Our work thus expands the repertoire of KRAB-ZFP/TRIM28 targets and, more importantly, reveals a new transcription factor shaping the embryonic epigenome by mediating regionally restricted germline-to-soma inheritance of epigenetic marks.

The inaccessibility of the early embryo to a directed *in vivo* approach for target gene identification is a common problem in the field. Ectopic overexpression of tagged ZFP708 (endogenously maternally restricted) in mESCs has proven a suitable approach for identifying RMER19B elements as putative targets. However, the extent of these interactions is to be taken with caution. For example, only a small fraction of all RMER19B elements in the genome were identified as true targets by peak calling, yet levels of enrichment can be observed across a much wider repertoire of RMER19B insertions (Fig. S3A). Conversely, binding to ‘other’ (non-repeat) regions (Fig. 2C) may be a result of the ectopic expression approach and might not reflect true endogenous targets, as suggested by the lack of H3K9me3 enrichment at such sites in ZFP708 OE mESCs and during development *in vivo* (Fig. 2E, Fig. S7F,G). Support for the selective targeting of a few RMER19B elements is provided by the H3K9me3 profiling in wild-type embryos via ultra-low ChIP-seq (Wang et al., 2018). It is evident that only a minor subset of RMER19B elements acquires H3K9me3 post-fertilization, a subset that shows a remarkable overlap with the ZFP708-bound fraction of RMER19B elements in ZFP708 OE mESCs (Fig. 4). Although the impact of ZFP708 binding to RMER19B elements in their entirety is not fully resolved, we were able to meticulously dissect the regional binding and transcriptional impact of ZFP708 on specific elements (Fig. 3). In this respect, ZFP708 functions as a canonical, TRIM28-recruiting KRAB-ZFP.

The interplay of DNA methylation and H3K9me3 at ZFP708 targets is unclear. This is true for all TRIM28-targeted regions in very early embryos described to date. Clearly, wild-type embryos retain DNA methylation at ZFP708-bound RMER19B elements (as shown in blastocysts and derived mESCs). This DNA methylation maintenance is dependent on maternal ZFP708, the lack of which results in severe hypomethylation (Fig. 5). In this respect, ZFP708 targets behave like genomic imprints or other rare genomic regions, such as the *Rbmy1a1* or the *Scml2* locus (Branco et al., 2016; Messerschmidt et al., 2012; Sampath Kumar et al., 2017). Gamete-inherited, regional DNA methylation is resistant to the intrinsic DNA demethylation drive of embryonic global epigenetic reprogramming. In contrast to the imprint maintaining ZFP57, binding of ZFP708 to DNA is DNA methylation independent (Fig. S4B-D).

Despite the epigenetic changes observed at ZFP708-bound RMER19B elements, the loss of ZFP708 itself does not affect viability or fertility, or cause any other overt phenotypes in mice. This lack of a phenotypic impact in ZFP708 knockout embryos, despite RMER19B hypomethylation, may suggest an archaic mechanism to repress an ERE family that has long since lost its mutagenic/regulatory potential. On the other hand, the lack of a detectable phenotype may merely reflect our limitations in gauging a possible transcriptional or evolutionary impact in a laboratory setting. Indeed, although the transcriptional effect of ZFP708 loss on RMER19B elements itself is not given in embryos, we find transcriptional changes of neighboring genes in mutant-derived mESCs and, to lesser extent, in embryos. These changes, in particular in embryos, are small and variable in individual embryos. On the other hand, the species-specific insertions of these RMER19B elements may drive the tissue-specific activation/repression of genes – an intriguing hypothesis to be tested in the future. Our current work however, provides insights into such potential regulatory functions, as ZFP708 overexpression in mESCs can alter RMER19B neighboring gene expression.

In support of the functional requirement of ZFP708, despite the apparent lack of a phenotypic effect when deleted, is the evolutionary conservation. Both RMER19B elements and ZFP708 are conserved at least in rats and mice, separated by near 20 million years of evolution. Other KRAB-ZFPs targeting EREs with stable inherent transcriptional activity and even retrotransposition potential have no discernible phenotypes if deleted *in vivo* either (Ecco et al., 2016; Wolf et al., 2015b). Furthermore, the potential impact of EREs on their host genomes may go beyond mere retrotransposition or transcriptional effects and cause subtle long-term effects or rare events of genome instability (reviewed by Ecco et al., 2017). And, last but not least, many EREs in mice and humans are targeted by multiple KRAB-ZFPs, often at divergent sites and possibly in a spatio-temporal context (Ecco et al., 2016; Imbeault et al., 2017). It is therefore conceivable that RMER19B elements may be retargeted for heterochromatinization at later developmental stages or in adult tissues by other KRAB-ZFPs.

It remains to be seen if and how such subtle, seemingly insignificant, disturbances in the early embryonic epigenome can upset the evolutionary trajectory of a species. The fact that RMER19B elements, and its repressive machinery and mechanisms are preserved throughout evolution in the first place, however, suggest that ZFP708 is there for a reason.

## MATERIALS AND METHODS

### Animal work, mESC derivation and cell lines

Mice were set up for natural mating and embryos were isolated as previously described (Behringer et al., 2013; Sampath Kumar et al., 2017). Zygotes were isolated at E (embryonic day) 0.5, two-cell stage embryos at E1.5, eight-cell stage embryos at E2.25-E2.5 and blastocysts at E3.5, assuming the time of mating being midnight of the mating day. Isolated embryos were immediately processed for RNA isolation or DNA methylation analysis. All animal experiments were conducted according to local IACUC regulations.

For mESC line derivation, E3.5 embryos were cultured on a MEF feeder layer in 2i medium. After 5-7 days, ICM outgrowths were passaged and cultured on MEF feeders with daily monitoring. mESC-like colonies were selected and gradually expanded. For routine propagation, cells were passaged every 2-3 days. For downstream applications, cells were adapted to feeder-free culture on gelatin-coated (0.1%) plates. DNMT TKO mESCs (clone 19-1; AES0146) were provided by the RIKEN BRC through the National Bio-Resource Project of the MEXT, Japan.

### ZFP708, and luciferase reporter cloning and expression

ZFP708 was cloned from ovary cDNA. N-terminal 3xFLAG-tagged ZFP708 or ZFP708-KΔ was produced by PCR amplification and cloning into the PJ549 piggy-bac vector (DNA2.0). 24 h after transfection, cells were selected with puromycin before GFP-positive cells were sorted by FACS. To test ZFP708-mediated repression, first the mouse *Pgk* promoter was cloned into the pGL4.23 vector (Promega) to drive luciferase activity. The *Uck2-RMER19B* (amplified from wild-type mESCs) or the *Uck2-ΔRMER19B* (amplified from CRISPR mutated mESC clone #15) were then cloned upstream of the *Pgk* promoter. HEK293T cells were transfected with *Uck2-RMER19B:Pgk-pGL4.23* or *Uck2-ΔRMER19B:Pgk-pGL4.23* reporter constructs along with FLAG-ZFP708 or FLAG-ZFP708-KΔ, respectively. Renilla activity, expressed from co-transfected pGL4.75 (Promega), was used for internal normalization. 48 h after transfection, cells were lysed and assayed using the Dual-Luciferase reporter assay system (Promega).

### Immunofluorescence

mESCs transfected with FLAG-ZFP708 were seeded on glass slides in 12-well plates to be fixed 48 h later with 4% formaldehyde and permeabilized with 0.1% Triton X-100. After blocking with FBS (10%), cells were incubated with mouse anti-FLAG (Sigma, 1:250) and rabbit anti-TRIM28 (Abcam, 1:250) antibodies. Secondary antibodies (Alexa Fluor 488 and 594, Invitrogen) were used at 1: 500 dilutions. Images were acquired on a Zeiss confocal laser-scanning microscope.

### Co-immunoprecipitation and western blot

Cells were lysed in RIPA buffer and protein was quantified using a Bradford protein assay (Bio-Rad). Lysates were analyzed by western blotting using the following antibodies: anti-Flag (Sigma, 1:5000), anti-TRIM28 (Abcam, 1:4000) and anti-tubulin (Sigma, 1:10,000). For co-immunoprecipitation analysis, the lysates were immunoprecipitated using anti-Trim28 antibody and analyzed for association with ZFP708 by western blot using anti-Flag antibody.

### Chromatin immunoprecipitation, sequencing and analysis

ChIP was performed as previously described (Guccione et al., 2007). Chromatin was fragmented using a BRANSON Digital Sonifier (#S540D) to 250-1000 bp fragments, precleared and incubated with antibodies overnight. Antibody/chromatin complexes were incubated with 50% protein-G sepharose slurry and washed before elution/decrosslinking in 1% SDS and 0.1 M NaHCO_3_. DNA was column purified with a QIAquick PCR Purification Kit (Qiagen).

ChIP DNA concentrations were measured using the Qubit dsDNA HS Assay Kit (Thermo Fisher Scientific). ChIP-seq libraries were prepared with the NEBNext Ultra II DNA Library Preparation Kit for Illumina (NEB), following manufacturer's instructions. PCR conditions were adjusted to starting material concentrations and final elution volumes were reduced. Libraries were quantified using a Bioanalyzer (High Sensitivity DNA Kit, Agilent) and PCR (KAPA Library Quantification Kit for Illumina, Roche). ChIP-Seq libraries were pooled and sequenced on the NextSeq500, as a single-end (75 bp) run.

Raw reads were aligned to the mm9 genome using Bowtie2 (default parameters), before sorting the bam files with samtools sort and indexing with samtools index. Bigwig files were generated with the bamCoverage function of deeptools2.0, using the options --normalizeUsing RPKM --binSize 25 --smoothLength 100. Peaks were called with Macs2 callpeak (default parameters). FLAG-ZFP708 peaks were called over a FLAG ChIP control (in wild-type mESCs) in three replicates and only peaks that were called in all three replicates were used for analysis. For heat maps, ChIP and input data were converted to bigwig files, normalized and corresponding inputs were subtracted. Heat maps, density profiles and violin plots were generated using Deeptools and GraphPad. The following published ChIP-seq datasets were used: FLAG-Zfp809 (SRX485302), TRIM28 (GSM1406445), SETDB1 (GSM440256), H3K9me3 (mESCs GSM1327148) and H3K9me3 (germ cells and embryos GSE97778). For peak overlap analysis TRIM28 ChIP-seq peaks in mESCs from Castro-Diaz et al. (2014), Quenneville et al. (2011), Riso et al. (2016) and Rowe et al. (2010) (GSM1406445, GSM1032198, GSM773067, GSM2051876/GSM2051868) were used. All overlaps were generated with BEDTools. MEME was used for motif analysis using standard settings.

### RNA isolation, RT-PCR, qRT-PCR from embryos, cells and tissues

Total RNA was isolated from cells and tissues using RNeasy Mini Kit (Qiagen) according to the manufacturer's instructions and 1 μg of total RNA was used for reverse transcription using the High-Capacity cDNA Kit (Applied Biosystems). Total RNA from pooled or individual embryos was isolated using PicoPure RNA Isolation Kit (Fisher Scientific) and all of the eluate was used for reverse transcription. Reverse transcription was performed using the High-Capacity cDNA Reverse Transcription Kit (Applied Biosystems) using random hexamer primers. qPCR assays for gene expression analysis were designed using the Assay Design Center Web Service (qpcr.probefinder.com/roche3.html, Roche Diagnostics). qPCR was performed using Taq-Man Fast Universal PCR Master Mix (Applied Biosystems) in combination with the universal probe library (Roche) on a CFX384 Touch Real Time PCR system (Bio-Rad). Relative gene expression (2^ΔCt^ method) was calculated using the housekeeping gene actin to normalize the target genes or as otherwise indicated.

### RNA-sequencing

RNA-seq libraries from single oocytes, zygotes and two-cell stage embryos were prepared largely following the single cell RNA-seq protocol developed by Picelli and colleagues with slight modifications (Picelli et al., 2014). The number of cycles for the initial cDNA amplification PCR was reduced to 16. The Nextera XT DNA Library Prep Kit (Illumina) was used to generate sequencing libraries as described previously (Picelli et al., 2014) with 1 ng of amplified cDNA. Resulting libraries were pooled and sequenced on a NextSeq500 instrument, at 1×75 bp single read length to >7 M reads per oocyte, zygote or embryo.

RNA-seq libraries from mESCs were generated using the NEBNext Ultra II Directional RNA Library Prep Kit for Illumina (NEB), after mRNA isolation using the NEBNext Poly(A) mRNA Magnetic Isolation Module (NEB) following manufacturer's instructions. The resulting libraries were pooled and sequenced on a NextSeq500 instrument, at a 1×75 bp single read length to >14 M reads per sample. Raw reads were aligned to the mm9 genome using STAR 2.4.2a [using options --sjdbGTFfile Mus_musculus.Ensembl.NCBIM37.65.gtf --sjdbOverhang $((lng - 1)) --outSAMstrandField intronMotif --outSAMtype BAM Unsorted --twopassMode Basic], before sorting the bam files with samtools sort and indexing with samtools index. The number of reads mapping to each gene was quantified with htseq-count (using options --order=pos --format=bam --stranded=reverse) on features from the gtf file Mus_musculus.Ensembl.NCBIM37.65.gtf. The raw counts of reads were then imported to DEseq2 in R to perform the differential expression analyses. The expression of transposable elements (TEs) was quantified using SalmonTE. The list of repeats was downloaded from Repbase (*Mus musculus* and ancestral) as a fasta file. This fasta file was then used to build the index on SalmonTE, using the --te_only option. The TEs expression was quantified with SalmonTE quant (default parameters) on the raw fastq files.

For ZFP708 expression analysis in germ cells and preimplantation embryos, we mined datasets GSE44183 and GSE66582 from Wu et al. (2016) and Xue et al. (2013). For illustration and comparison across samples and studies, expression levels were normalized to actin*.*

### Mutagenesis

gRNAs were designed using E-CRISP (e-crisp.org) (Heigwer et al., 2014) and oligonucleotides were cloned into PX458 as described previously (Ran et al., 2013). For mESC, GFP-positive cells were FACS sorted into 96-well plates and clonally expanded before sequencing. For indel knockout mice, zygotes were injected with CAS9 cRNA and gRNAs before retransfer into pseudopregnant females. DNA was extracted from pup tail biopsies and targeted regions were cloned and sequenced to identify founder lines.

### DNA methylation analysis by bisulfite conversion/sequencing and COBRA

DNA methylation was analyzed by bisulfite conversion, cloning and sequencing as described previously (Messerschmidt et al., 2012). Briefly, DNA from one pooled litter of E3.5 embryos (minimum eight embryos) was used for bisulfite conversion according to the manufacturer's protocol (Imprint DNA Modification Kit, Sigma). For sperm and mESC samples 1 μg of genomic DNA was used for conversion. Converted DNA was eluted in 20 µl and 1 µl (sperm, mESCs) or 5 µl (blastocysts) and used for PCR. PCR fragments were cloned and analyzed by Sanger sequencing. COBRA assays were performed as previously described (Messerschmidt et al., 2012).

### Oligonucleotides and antibodies used

Oligonucleotides used in this study are listed in Table S14. All information for antibodies used in this study is listed in Table S15.

## Supplementary Material

Supplementary information

## References

[DEV170266C1] Behringer R., Gertsenstein M., Nagy K. V. and Nagy A. (2013). *Manipulating the Mouse Embryo: A Laboratory Manual*. Cold Spring Harbor Laboratory Press.

[DEV170266C2] Branco M. R., King M., Perez-Garcia V., Bogutz A. B., Caley M., Fineberg E., Lefebvre L., Cook S. J., Dean W., Hemberger M. et al. (2016). Maternal DNA methylation regulates early trophoblast development. *Dev. Cell* 36, 152-163. 10.1016/j.devcel.2015.12.027 26812015 PMC4729543

[DEV170266C3] Castro-Diaz N., Ecco G., Coluccio A., Kapopoulou A., Yazdanpanah B., Friedli M., Duc J., Jang S. M., Turelli P. and Trono D. (2014). Evolutionally dynamic L1 regulation in embryonic stem cells. *Genes Dev.* 28, 1397-1409. 10.1101/gad.241661.114 24939876 PMC4083085

[DEV170266C4] Cirio M. C., Ratnam S., Ding F., Reinhart B., Navara C. and Chaillet J. R. (2008). Preimplantation expression of the somatic form of Dnmt1 suggests a role in the inheritance of genomic imprints. *BMC Dev. Biol.* 8, 9 10.1186/1471-213X-8-9 18221528 PMC2266903

[DEV170266C5] Dan J., Liu Y., Liu N., Chiourea M., Okuka M., Wu T., Ye X., Mou C., Wang L., Wang L. et al. (2014). Rif1 maintains telomere length homeostasis of ESCs by mediating heterochromatin silencing. *Dev. Cell* 29, 7-19. 10.1016/j.devcel.2014.03.004 24735877 PMC4720134

[DEV170266C6] Ecco G., Cassano M., Kauzlaric A., Duc J., Coluccio A., Offner S., Imbeault M., Rowe H. M., Turelli P. and Trono D. (2016). Transposable elements and their KRAB-ZFP controllers regulate gene expression in adult tissues. *Dev. Cell* 36, 611-623. 10.1016/j.devcel.2016.02.024 27003935 PMC4896391

[DEV170266C7] Ecco G., Imbeault M. and Trono D. (2017). KRAB zinc finger proteins. *Development* 144, 2719-2729. 10.1242/dev.132605 28765213 PMC7117961

[DEV170266C8] Emerson R. O. and Thomas J. H. (2009). Adaptive evolution in zinc finger transcription factors. *PLoS Genet.* 5, e1000325 10.1371/journal.pgen.1000325 19119423 PMC2604467

[DEV170266C9] Friedman J. R., Fredericks W. J., Jensen D. E., Speicher D. W., Huang X. P., Neilson E. G. and Rauscher F. J. (1996). KAP-1, a novel corepressor for the highly conserved KRAB repression domain. *Genes Dev.* 10, 2067-2078. 10.1101/gad.10.16.2067 8769649

[DEV170266C10] Guccione E., Bassi C., Casadio F., Martinato F., Cesaroni M., Schuchlautz H., Lüscher B. and Amati B. (2007). Methylation of histone H3R2 by PRMT6 and H3K4 by an MLL complex are mutually exclusive. *Nature* 449, 933-937. 10.1038/nature06166 17898714

[DEV170266C11] Heigwer F., Kerr G. and Boutros M. (2014). E-CRISP: fast CRISPR target site identification. *Nat. Methods* 11, 122-123. 10.1038/nmeth.2812 24481216

[DEV170266C12] Hirasawa R., Chiba H., Kaneda M., Tajima S., Li E., Jaenisch R. and Sasaki H. (2008). Maternal and zygotic Dnmt1 are necessary and sufficient for the maintenance of DNA methylation imprints during preimplantation development. *Genes Dev.* 22, 1607-1616. 10.1101/gad.1667008 18559477 PMC2428059

[DEV170266C13] Imbeault M., Helleboid P.-Y. and Trono D. (2017). KRAB zinc-finger proteins contribute to the evolution of gene regulatory networks. *Nature* 543, 550-554. 10.1038/nature21683 28273063

[DEV170266C14] Jacobs F. M. J., Greenberg D., Nguyen N., Haeussler M., Ewing A. D., Katzman S., Paten B., Salama S. R. and Haussler D. (2014). An evolutionary arms race between KRAB zinc-finger genes ZNF91/93 and SVA/L1 retrotransposons. *Nature* 516, 242-245. 10.1038/nature13760 25274305 PMC4268317

[DEV170266C15] Kauzlaric A., Ecco G., Cassano M., Duc J., Imbeault M. and Trono D. (2017). The mouse genome displays highly dynamic populations of KRAB-zinc finger protein genes and related genetic units. *PLoS ONE* 12, e0173746 10.1371/journal.pone.0173746 28334004 PMC5363842

[DEV170266C16] Krebs C. J., Larkins L. K., Khan S. M. and Robins D. M. (2005). Expansion and diversification of KRAB zinc-finger genes within a cluster including Regulator of sex-limitation 1 and 2. *Genomics* 85, 752-761. 10.1016/j.ygeno.2005.03.004 15885501

[DEV170266C17] Krebs C. J., Khan S., MacDonald J. W., Sorenson M. and Robins D. M. (2009). Regulator of sex-limitation KRAB zinc finger proteins modulate sex-dependent and -independent liver metabolism. *Physiol. Genomics* 38, 16-28. 10.1152/physiolgenomics.90391.2008 19351907 PMC2696148

[DEV170266C18] Kurihara Y., Kawamura Y., Uchijima Y., Amamo T., Kobayashi H., Asano T. and Kurihara H. (2008). Maintenance of genomic methylation patterns during preimplantation development requires the somatic form of DNA methyltransferase 1. *Dev. Biol.* 313, 335-346. 10.1016/j.ydbio.2007.10.033 18048024

[DEV170266C19] Li X., Ito M., Zhou F., Youngson N., Zuo X., Leder P. and Ferguson-Smith A. C. (2008). A maternal-zygotic effect gene, Zfp57, maintains both maternal and paternal imprints. *Dev. Cell* 15, 547-557. 10.1016/j.devcel.2008.08.014 18854139 PMC2593089

[DEV170266C20] Liu Y., Toh H., Sasaki H., Zhang X. and Cheng X. (2012). An atomic model of Zfp57 recognition of CpG methylation within a specific DNA sequence. *Genes Dev.* 26, 2374-2379. 10.1101/gad.202200.112 23059534 PMC3489995

[DEV170266C21] Liu H., Chang L.-H., Sun Y., Lu X. and Stubbs L. (2014). Deep vertebrate roots for mammalian zinc finger transcription factor subfamilies. *Genome Biol. Evol.* 6, 510-525. 10.1093/gbe/evu030 24534434 PMC3971581

[DEV170266C22] Lorthongpanich C., Doris T. P. Y., Limviphuvadh V., Knowles B. B. and Solter D. (2012). Developmental fate and lineage commitment of singled mouse blastomeres. *Development* 139, 3722-3731. 10.1242/dev.086454 22991438

[DEV170266C23] Lorthongpanich C., Cheow L. F., Balu S., Quake S. R., Knowles B. B., Burkholder W. F., Solter D. and Messerschmidt D. M. (2013). Single-cell DNA-methylation analysis reveals epigenetic chimerism in preimplantation embryos. *Science* 341, 1110-1112. 10.1126/science.1240617 24009393

[DEV170266C24] Macfarlan T. S., Gifford W. D., Driscoll S., Lettieri K., Rowe H. M., Bonanomi D., Firth A., Singer O., Trono D. and Pfaff S. L. (2012). Embryonic stem cell potency fluctuates with endogenous retrovirus activity. *Nature* 487, 57-63. 10.1038/nature11244 22722858 PMC3395470

[DEV170266C25] Messerschmidt D. M., de Vries W., Ito M., Solter D., Ferguson-Smith A. and Knowles B. B. (2012). Trim28 is required for epigenetic stability during mouse oocyte to embryo transition. *Science* 335, 1499-1502. 10.1126/science.1216154 22442485

[DEV170266C26] Messerschmidt D. M., Knowles B. B. and Solter D. (2014). DNA methylation dynamics during epigenetic reprogramming in the germline and preimplantation embryos. *Genes Dev.* 28, 812-828. 10.1101/gad.234294.113 24736841 PMC4003274

[DEV170266C27] Peaston A. E., Evsikov A. V., Graber J. H., de Vries W. N., Holbrook A. E., Solter D. and Knowles B. B. (2004). Retrotransposons regulate host genes in mouse oocytes and preimplantation embryos. *Dev. Cell* 7, 597-606. 10.1016/j.devcel.2004.09.004 15469847

[DEV170266C28] Picelli S., Faridani O. R., Björklund A. K., Winberg G., Sagasser S. and Sandberg R. (2014). Full-length RNA-seq from single cells using Smart-seq2. *Nat. Protoc.* 9, 171-181. 10.1038/nprot.2014.006 24385147

[DEV170266C29] Quenneville S., Verde G., Corsinotti A., Kapopoulou A., Jakobsson J., Offner S., Baglivo I., Pedone P. V., Grimaldi G., Riccio A. et al. (2011). In embryonic stem cells, ZFP57/KAP1 recognize a methylated hexanucleotide to affect chromatin and DNA methylation of imprinting control regions. *Mol. Cell* 44, 361-372. 10.1016/j.molcel.2011.08.032 22055183 PMC3210328

[DEV170266C30] Quenneville S., Turelli P., Bojkowska K., Raclot C., Offner S., Kapopoulou A. and Trono D. (2012). The KRAB-ZFP/KAP1 system contributes to the early embryonic establishment of site-specific DNA methylation patterns maintained during development. *Cell Rep.* 2, 766-773. 10.1016/j.celrep.2012.08.043 23041315 PMC3677399

[DEV170266C31] Ran F. A., Hsu P. D., Wright J., Agarwala V., Scott D. A. and Zhang F. (2013). Genome engineering using the CRISPR-Cas9 system. *Nat. Protoc.* 8, 2281-2308. 10.1038/nprot.2013.143 24157548 PMC3969860

[DEV170266C32] Riso V., Cammisa M., Kukreja H., Anvar Z., Verde G., Sparago A., Acurzio B., Lad S., Lonardo E., Sankar A. et al. (2016). ZFP57 maintains the parent-of-origin-specific expression of the imprinted genes and differentially affects non-imprinted targets in mouse embryonic stem cells. *Nucleic Acids Res.* 44, 8165-8178. 10.1093/nar/gkw505 27257070 PMC5041456

[DEV170266C33] Rowe H. M., Jakobsson J., Mesnard D., Rougemont J., Reynard S., Aktas T., Maillard P. V., Layard-Liesching H., Verp S., Marquis J. et al. (2010). KAP1 controls endogenous retroviruses in embryonic stem cells. *Nature* 463, 237-240. 10.1038/nature08674 20075919

[DEV170266C34] Rowe H. M., Friedli M., Offner S., Verp S., Mesnard D., Marquis J., Aktas T. and Trono D. (2013). De novo DNA methylation of endogenous retroviruses is shaped by KRAB-ZFPs/KAP1 and ESET. *Development* 140, 519-529. 10.1242/dev.087585 23293284 PMC4892343

[DEV170266C35] Sampath Kumar A., Seah M. K. Y., Ling K. Y., Wang Y., Tan J. H. L., Nitsch S., Lim S. L., Lorthongpanich C., Wollmann H., Low D. H. P. et al. (2017). Loss of maternal Trim28 causes male-predominant early embryonic lethality. *Genes Dev.* 31, 12-17. 10.1101/gad.291195.116 28115466 PMC5287108

[DEV170266C36] Schultz D. C., Friedman J. R. and Rauscher F. J. (2001). Targeting histone deacetylase complexes via KRAB-zinc finger proteins: the PHD and bromodomains of KAP-1 form a cooperative unit that recruits a novel isoform of the Mi-2alpha subunit of NuRD. *Genes Dev.* 15, 428-443. 10.1101/gad.869501 11230151 PMC312636

[DEV170266C37] Schultz D. C., Ayyanathan K., Negorev D., Maul G. G. and Rauscher F. J. (2002). SETDB1: a novel KAP-1-associated histone H3, lysine 9-specific methyltransferase that contributes to HP1-mediated silencing of euchromatic genes by KRAB zinc-finger proteins. *Genes Dev.* 16, 919-932. 10.1101/gad.973302 11959841 PMC152359

[DEV170266C38] Thomas J. H. and Schneider S. (2011). Coevolution of retroelements and tandem zinc finger genes. *Genome Res.* 21, 1800-1812. 10.1101/gr.121749.111 21784874 PMC3205565

[DEV170266C39] Urrutia R. (2003). KRAB-containing zinc-finger repressor proteins. *Genome Biol.* 4, 231 10.1186/gb-2003-4-10-231 14519192 PMC328446

[DEV170266C40] Wang C., Liu X., Gao Y., Yang L., Li C., Liu W., Chen C., Kou X., Zhao Y., Chen J. et al. (2018). Reprogramming of H3K9me3-dependent heterochromatin during mammalian embryo development. *Nat. Cell Biol.* 20, 620-631. 10.1038/s41556-018-0093-4 29686265

[DEV170266C41] Wiznerowicz M., Jakobsson J., Szulc J., Liao S., Quazzola A., Beermann F., Aebischer P. and Trono D. (2007). The Kruppel-associated box repressor domain can trigger de novo promoter methylation during mouse early embryogenesis. *J. Biol. Chem.* 282, 34535-34541. 10.1074/jbc.M705898200 17893143

[DEV170266C42] Wolf G., Greenberg D. and Macfarlan T. S. (2015a). Spotting the enemy within: targeted silencing of foreign DNA in mammalian genomes by the Krüppel-associated box zinc finger protein family. *Mobile DNA* 6, 17. 26435754 10.1186/s13100-015-0050-8 PMC4592553

[DEV170266C43] Wolf G., Yang P., Füchtbauer A. C., Füchtbauer E.-M., Silva A. M., Park C., Wu W., Nielsen A. L., Pedersen F. S. and Macfarlan T. S. (2015b). The KRAB zinc finger protein ZFP809 is required to initiate epigenetic silencing of endogenous retroviruses. *Genes Dev.* 29, 538-554. 10.1101/gad.252767.114 25737282 PMC4358406

[DEV170266C44] Wu J., Huang B., Chen H., Yin Q., Liu Y., Xiang Y., Zhang B., Liu B., Wang Q., Xia W. et al. (2016). The landscape of accessible chromatin in mammalian preimplantation embryos. *Nature* 534, 652-657. 10.1038/nature18606 27309802

[DEV170266C45] Xue Z., Huang K., Cai C., Cai L., Jiang C.-Y., Feng Y., Liu Z., Zeng Q., Cheng L., Sun Y. E. et al. (2013). Genetic programs in human and mouse early embryos revealed by single-cell RNA sequencing. *Nature* 500, 593-597. 10.1038/nature12364 23892778 PMC4950944

[DEV170266C46] Yang P., Wang Y., Hoang D., Tinkham M., Patel A., Sun M.-A., Wolf G., Baker M., Chien H.-C., Lai K.-Y. N. et al. (2017). A placental growth factor is silenced in mouse embryos by the zinc finger protein ZFP568. *Science* 356, 757-759. 10.1126/science.aah6895 28522536 PMC6309218

[DEV170266C47] Yuan P., Han J., Guo G., Orlov Y. L., Huss M., Loh Y.-H., Yaw L.-P., Robson P., Lim B. and Ng H.-H. (2009). Eset partners with Oct4 to restrict extraembryonic trophoblast lineage potential in embryonic stem cells. *Genes Dev.* 23, 2507-2520. 10.1101/gad.1831909 19884257 PMC2779752

