# Supplementary material for: The KRAB-zinc-finger protein ZFP708 mediates epigenetic repression at RMER19B retrotransposons
Source: Development. 2019 Jul 10;146(19):dev170266. doi: 10.1242/dev.170266 (PMC6803371; doi:10.1242/dev.170266)
Supplement: Supplementary information [file develop-146-170266-s1.pdf]

A

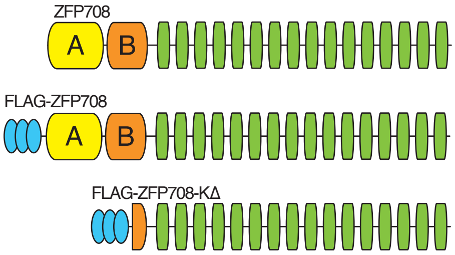

B

```
MIIEVFINAKEMTKDPLSFTDVAIDFSADECECLDSAQWNLYKEVMLENYSN
LVFLGLAFSKPYLVTFLEQSSDLWSVKRKASVAIHLGSKAHKCKECGKSFDR
NSVLIQHQRIHTGERPYKCEECGMAFTGRCILSKHQRIHTGEKPYKCKECGK
AFSCSSNLHQHQQIHREEKPYKCDDCGKAFSCSSYLYKHQRIHTGMKPYKCK
ECGKAFNCSANLIYHQRIHTGEKPYKCGECGKAFTIYSTFMNHQRTHSGEKP
YECKDCGKAFTNCYNLIQHQRIHTGEKPYACEECGKSFNYSSHLKQHKRIHT
GEKPYKCEVCGKAFNCSDYLVKHQRIHTGEKPYKCEVCGKAFSFSTYLHKHQ
RIHTGEKRYRCEECGKAFTNYSGLIVHRRVHTGEKPYKCEECGKAFSVHTSL
SKHQRIHTREQPYKCDECGKTFSVHSTFSKHQIMHTREKPYKCKHCDKTFKY
PSPLVQHERIHTGERPYKCEVCGKAFTSSSNLKYHWRLHTGEKPYKCEQCGK
AFKNSSNVTRHYKIHTRENPD
```

Figure S1. The KRAB-Zinc Finger Protein ZFP708.

(A) Schematic illustration of ZFP708 (top), 3xFLAG-ZFP708 (middle) and 3xFLAG-ZFP708-KΔ (bottom) (B) Protein sequence of the longest annotated *Zfp708* isoform. ZFP708 contains a typical N-terminal KRAB-domain consisting of a KRAB-A (yellow) and a KRAB-B (orange) box. The KRAB-domain is followed by 16 classical C2H2-Zinc fingers (green boxes). Grey amino acid residues were deleted for a TRIM28-interaction incompetent FLAG-ZFP708-KΔ version losing the whole KRAB-A and partial KRAB-B box.

Development • Supplementary information

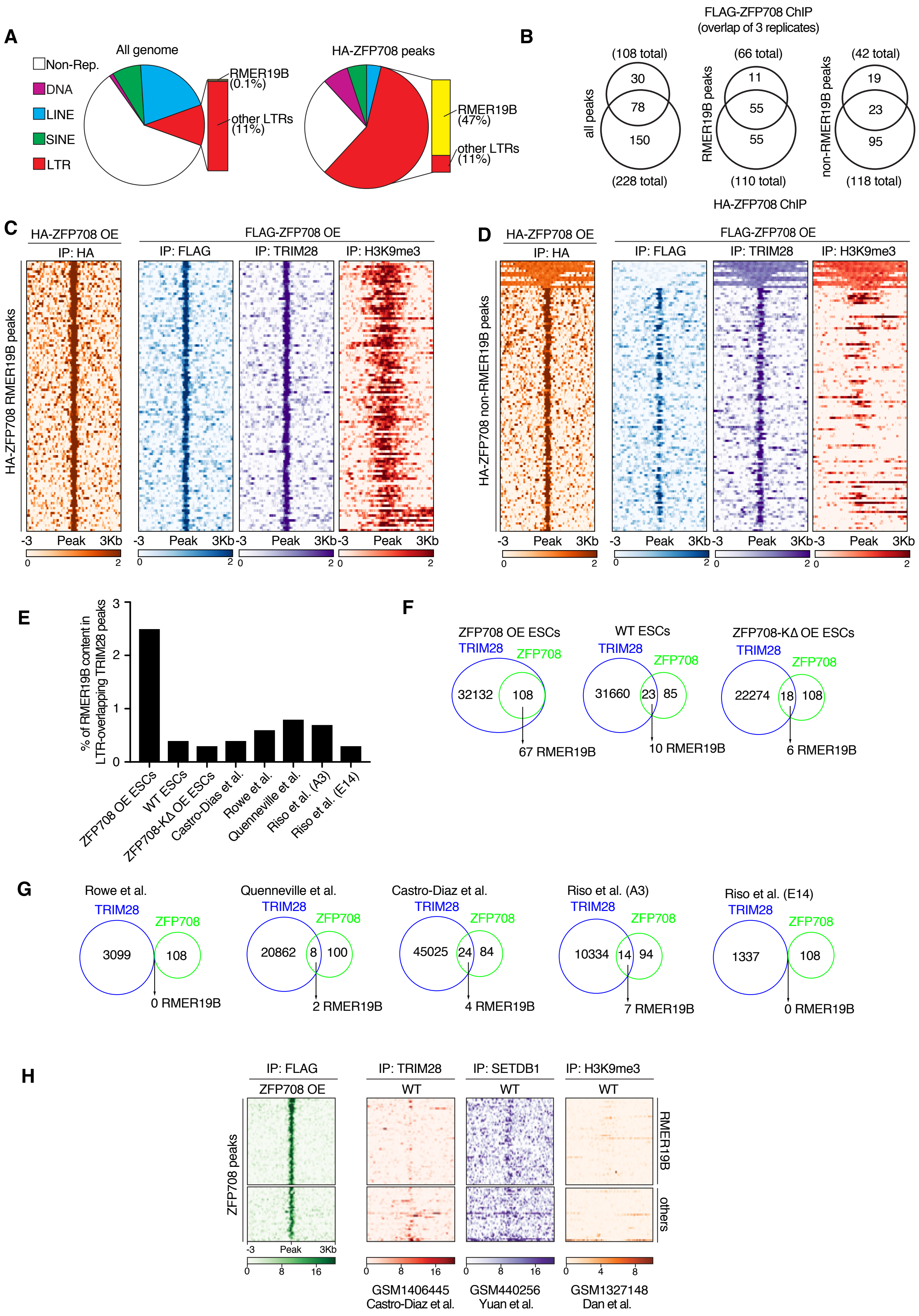

Development • Supplementary information

Figure S2. HA-ZFP708 ChIP-seq analysis and peak comparison with public datasets. (A) Distribution of genomic features across whole genome and identified HA-ZFP708 ChIP-seq peaks, respectively. (B) Venn diagrams depicting the overlaps of HA-ZFP708 and FLAG-ZFP708 (common peaks across three independent experiments) ChIP-seq peaks (all peaks and subcategorized into RMER19B-overlapping and non-RMER19B-overlapping peaks, from left to right respectively). (C) Heat map of HA enrichment at RMER19B HA-ZFP708 ChIP-seq peak regions in HA-ZFP708 OE mESCs and corresponding regions in FLAG-ZFP708 OE mESCs for FLAG, TRIM28 and H3K9me3 enrichment. (D) Same analysis as shown in (C) yet non-RMER19B HA-ZFP708 ChIP-seq peak regions are depicted. (E) Percentage of RMER19B retrotransposons found among the TRIM28-bound LTR retrotransposons in our own and published mESC TRIM28 ChIP datasets. (F) Overlap of TRIM28 and FLAG-ZFP708 ChIP-seq peaks in FLAG-ZFP708 OE, wildtype and ZFP708-KΔ OE mESCs. (G) Overlap of TRIM28 and FLAG-ZFP708 ChIP-seq peaks in five publically available datasets. (H) Heat map of FLAG enrichment at FLAG-ZFP708 ChIP-seq peak regions in FLAG-ZFP708 OE mESCs is compared to published TRIM28 (GSM1406445), SETDB1 (GSM440256) and H3K9me3 (GSM1327148) ChIP-seq data from wildtype mESCs. In all heat maps FLAG-ZFP708 peak summits are centered and 3Kb regions up- and downstream are displayed.

Development • Supplementary information

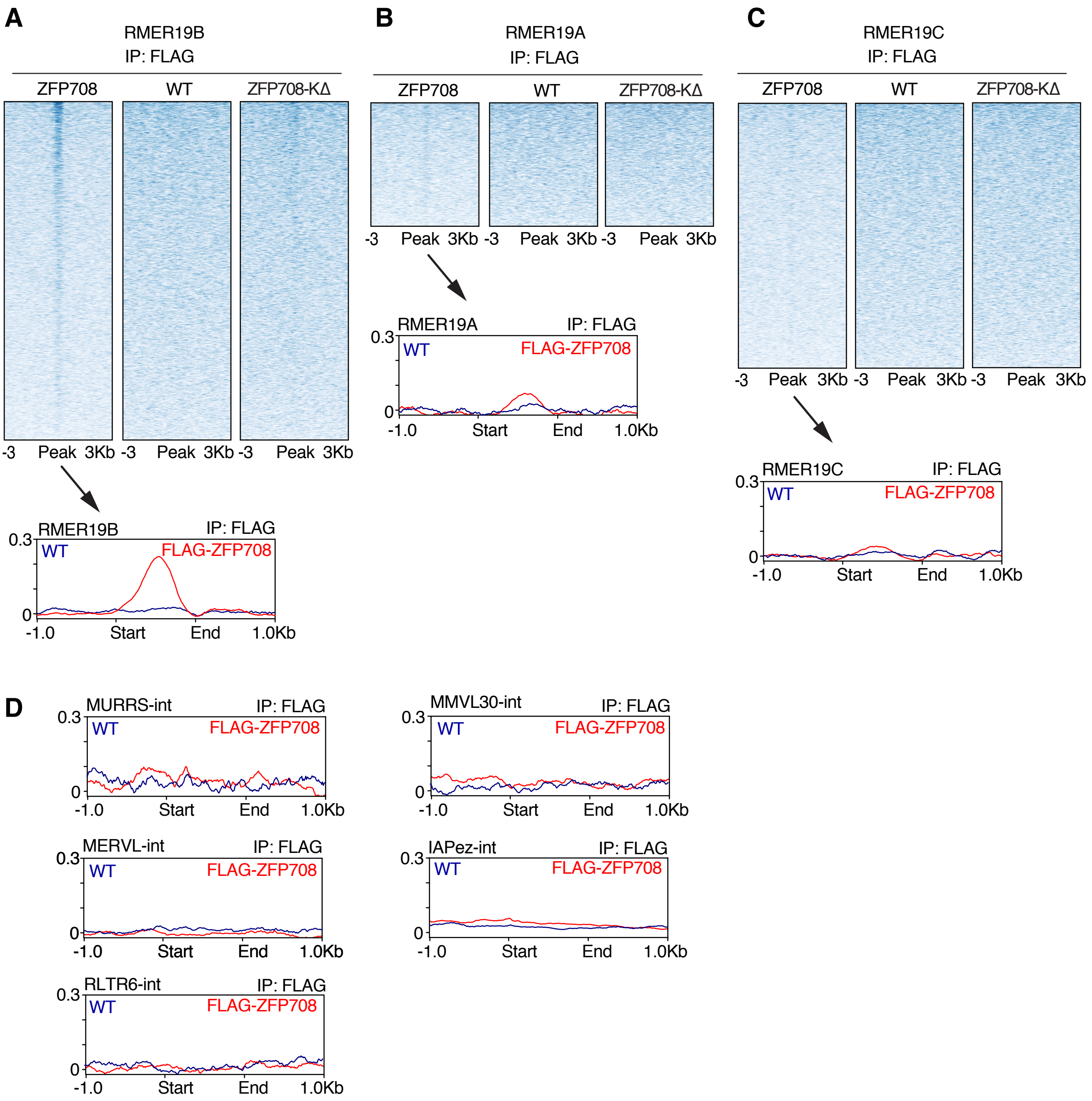

Development • Supplementary information

Figure S3. ZFP708 enrichment over related RMER19 family members and other EREs (A-C) Heat maps and density plots showing FLAG enrichment over RMER19B, RMER19A and RMER19C elements, respectively. Heat maps for FLAG-ZFP708 OE (note extensive RMER19B binding beyond the high-confidence triple overlap targets described in main text), wildtype and ZFP708-KΔ OE mESCs are shown. Peak regions were centered and 3Kb regions up- and downstream are displayed. Density plots below heat maps compare FLAG enrichment in FLAG-ZFP708 OE an wildtype ESCs across all elements of the respective RMER19 families. EREs were fitted to 1Kb in length additional 1Kb regions up- and downstream are displayed. (D) Same density plot analysis as described in (A) across other EREs. Only RMER19B elements show FLAG-ZFP708 enrichment.

Development • Supplementary information

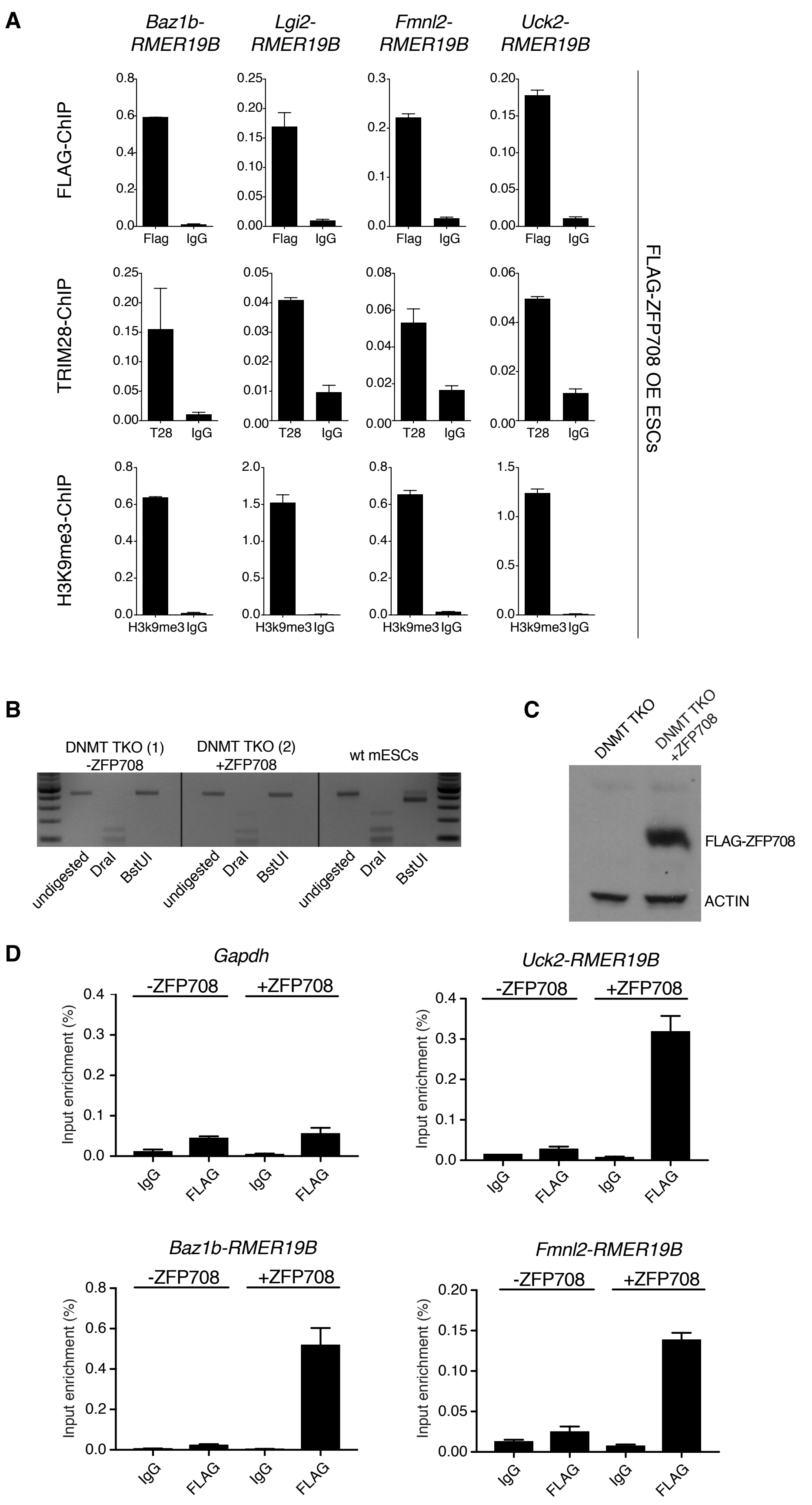

Development • Supplementary information

Figure S4. ChIP-seq confirmation by ChIP-qPCR and DNA methylation-independent binding of ZFP708 to DNA.

(A) Four FLAG-ZFP708 peak regions identified by ChIP-seq were selected for independent ChIP-qPCR confirmation. Enrichment at these sites was confirmed for FLAG-, TRIM28-, and H3K9me3 ChIP in FLAG-ZFP708 OE mESCs (n=3 technical replicates; error bars represent standard deviation of the mean; one representative of n=3 independent experiments is shown) (B) DNA methylation analysis for the H19 imprinting control region as a representative locus in two DNMT TKO and a control mESC line. DNA methylation is absent in DNMT TKO cells. (C) Western blot analysis of DNMT TKO mESC line with and without overexpression of FLAG-ZFP708. (D) ChIP-qPCR analysis for three FLAG-ZFP708 binding sites and a negative control (*Gapdh*) in DNMT TKO mESCs with and without stable FLAG-ZFP708 expression. FLAG ChIP shows strong enrichment for target sites in FLAG-ZFP708 OE DNMT TKO mESCs and in absence of DNA methylation. (n=2 technical replicates; error bars represent standard deviation of the mean; one representative of three independent experiments is shown).

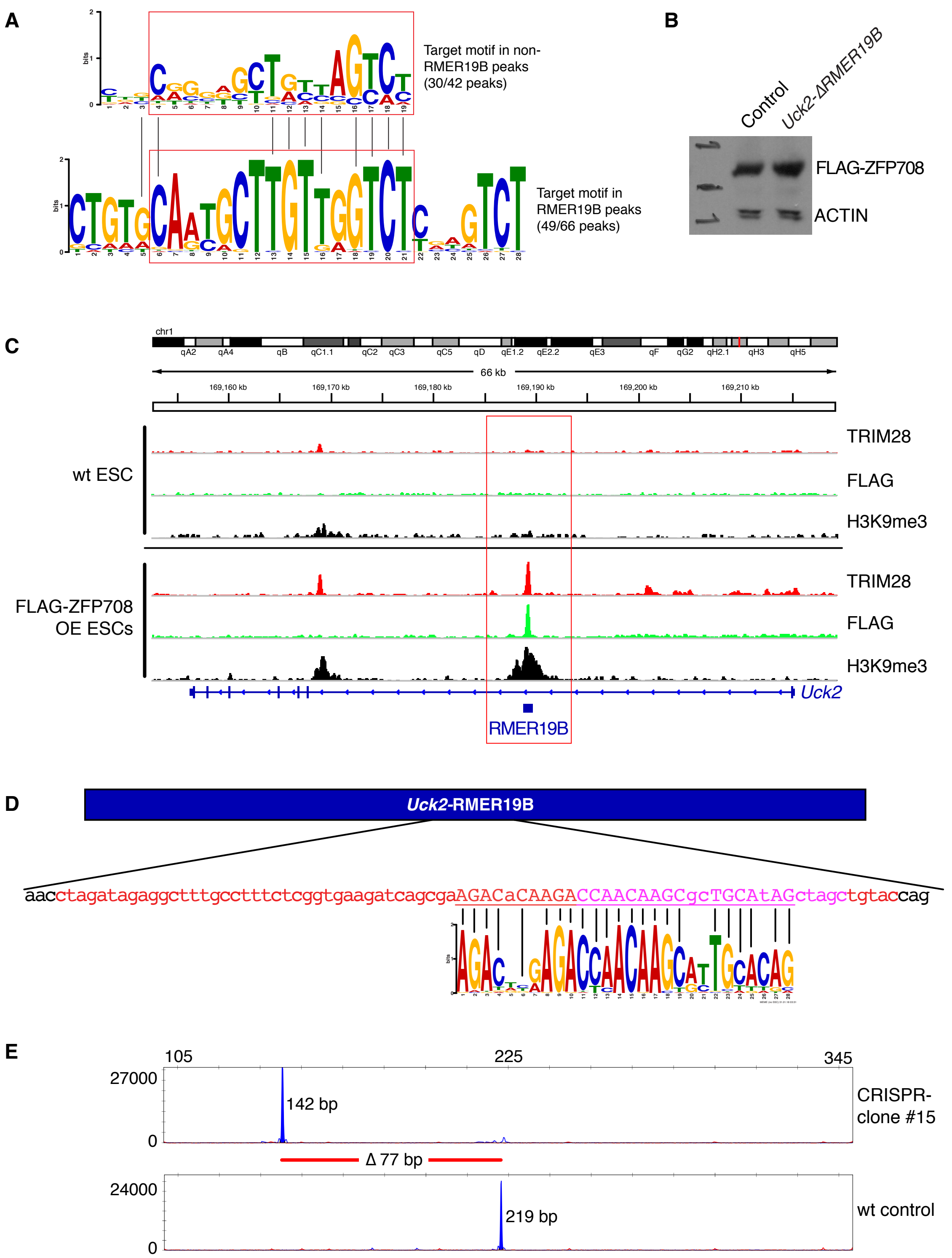

Development • Supplementary information

Figure S5. ZFP708 binding site prediction and deletion at the *Uck2*-RMER19B locus. (A) Full length predicted motifs of ZFP708 binding underlying non-RMER19B (top) and RMER19B (bottom) ChIP-seq peaks. Boxed cores are shown in main Figure 3A. (B) FLAG-ZFP708 expression verification in both control and *Uck2*-ΔRMER19B ESC lines by western blot. Actin serves as loading control. (C) Genome browser view of ZFP708, Trim28 and H3K9me3 enrichment in wildtype and FLAG-ZFP708 OE ESCs at the *Uck2* gene locus. The RMER19B integration in the first intron of *Uck2* is highlighted (red box). (D) Schematic illustration of the *Uck2*-RMER19B with predicted binding site and comparison with the identified ZFP708-binding motif (in reverse complement). Nucleotides in capital are highly conserved, nucleotides in red (77bp) are deleted in the *Uck2*-ΔRMER19B ESC clone, nucleotides in magenta are complementary to the Crispr guide RNA used for mutagenesis. (E) Gene-scan analysis of the *Crispr* indel mutation for clone #15. This clone carries a homozygous 77bp base pair deletion.

Development • Supplementary information

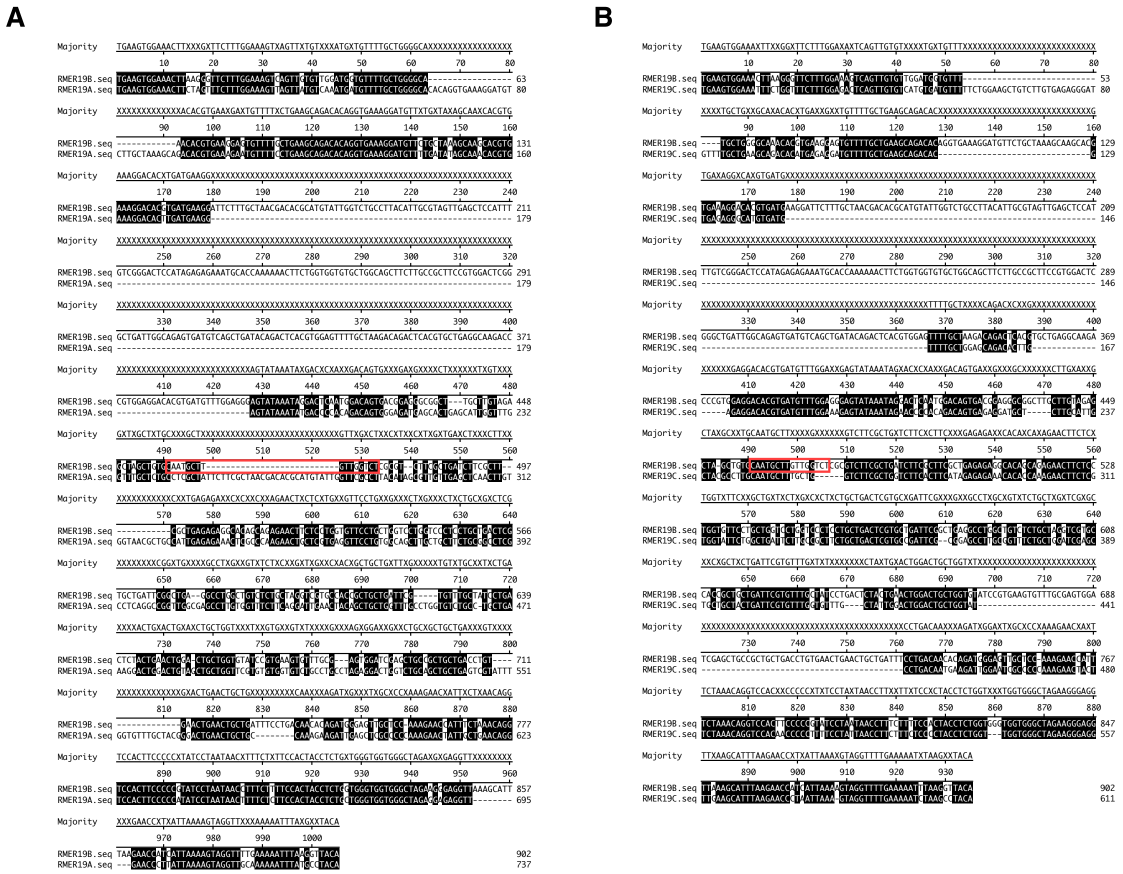

Figure S6. Pairwise sequence alignments of RMER19B and RMER19A/C EREs. Pairwise DNA alignment of (A) RMER19B/RMER19A or (B) RMER19B/RMER19C consensus sequences. The identified putative ZFP708 binding motif is boxed in red. Neither RMER19A nor RMER19C show full conservation of this site.

Development • Supplementary information

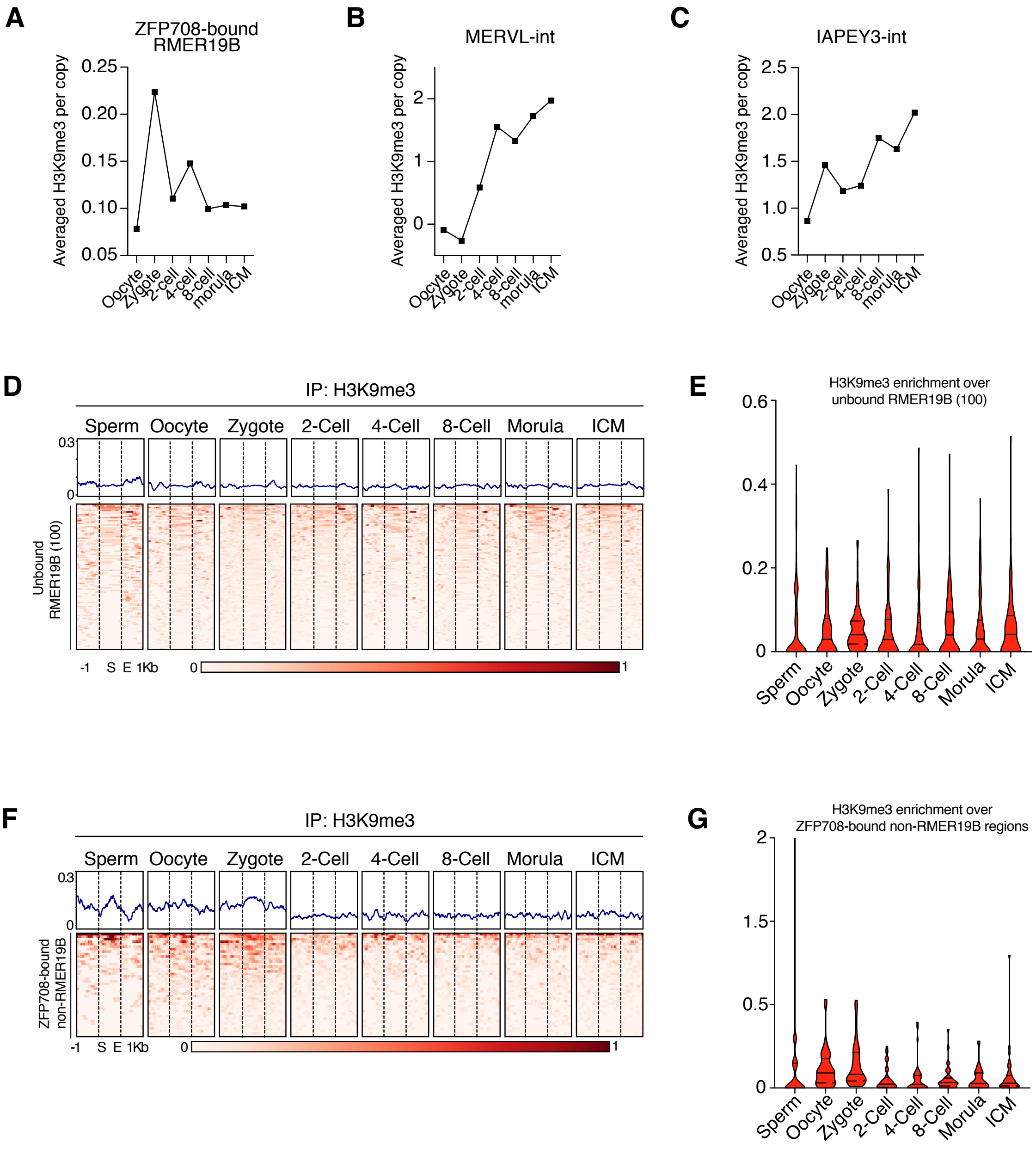

Development • Supplementary information

Figure S7. ZFP708 and RMER19B H3K9me3 analysis throughout preimplantation development

(A-C) Averaged H3K9me3 enrichment in oocytes and preimplantation embryos across ZFP708-bound RMER19B elements (A), MERVL-int (B) and IAPEY3-int elements (C). (D) Heat maps depicting H3K9me3 enrichment over 100 ZFP708-unbound RMER19B elements in sperm, oocytes and preimplantation embryos. EREs were fitted to 1Kb in length and additional 1Kb regions up- and downstream are displayed. (E) Violin plots quantifying the H3K9me3 enrichment across the RMER19B elements displayed in (D) omitting the flanking regions. (F) Heat maps depicting H3K9me3 enrichment over ZFP708-bound non-RMER19B peak regions in sperm, oocytes and preimplantation embryos. EREs were fitted to 1Kb in length and additional 1Kb regions up- and downstream are displayed. (G) Violin plots quantifying the H3K9me3 enrichment across the RMER19B elements displayed in (F) omitting the flanking regions.

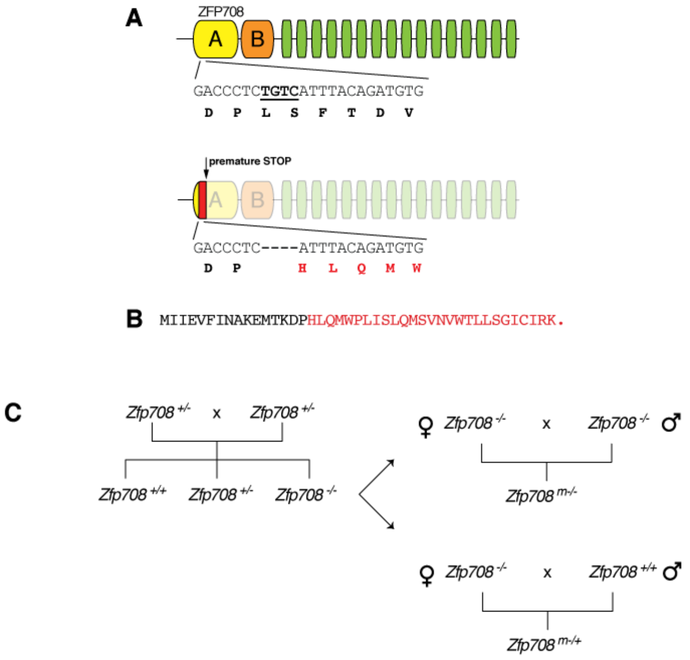

Figure S8. *Crisr*/CAS9 indel mutation of *Zfp708 in-vivo* and mating scheme. (A) Schematic illustration of wildtype (top) and predicted mutant (bottom) ZFP708 after *Crispr* mutation. Yellow and orange boxes represent KRAB-A and KRAB-B boxes respectively; green boxes represent predicted C2H2 zinc fingers. *Crispr* mutation caused a four nucleotide deletion (bold and underlined) creating a frame shift mutation (amino acid residues after frame-shift shown in red) followed by a premature translational stop codon. (B) Sequence of the predicted truncated ZFP708 protein produced from the *ZFP*708 locus after *Crispr*-mediated deletion of the four nucleotides indicated in (A). (C) Heterozygous intercross of ZFP780$^{+/-}$ mice give rise to viable and fertile ZFP708$^{-/-}$ (zygotic null) animals. Breeding ZFP708$^{-/-}$ (zygotic null) females to ZFP708$^{-/-}$ (zygotic null) males will give rise to ZFP708$^{m-/-}$ (maternal/zygotic null) animals. Breeding ZFP708$^{-/-}$ (zygotic null) females to ZFP708$^{+/+}$ (wildtype) males will give rise to ZFP708$^{m-/+}$ (maternal null) animals.

Development • Supplementary information

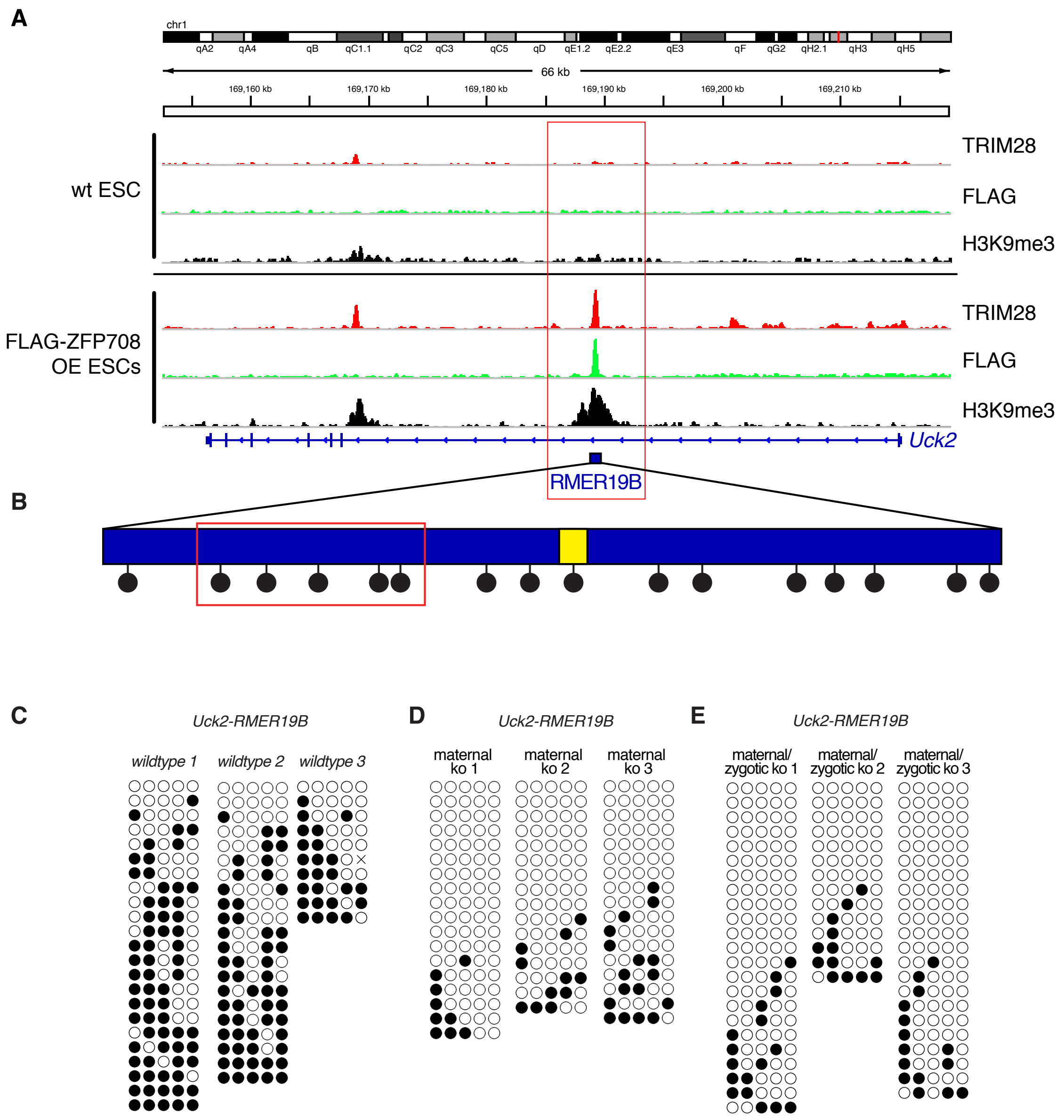

Development • Supplementary information

Figure S9. DNA methylation analysis at the *Uck2-RMER19B* locus

(A) Genome browser view of ZFP708, TRIM28 and H3K9me3 enrichment in wildtype and FLAG-ZFP708 OE ESCs at the *Uck2* gene locus. The RMER19B integration in the first intron of *Uck2* is highlighted (red box). (B) Schematic representation of the *Uck2-RMER19B.* The binding site is labelled in yellow, CpG sites are indicated as lollipops, the analyzed CpGs are boxed in red. (C-E) DNA methylation state at the indicated CpGs in three independently derived ESCs lines from wildtype (C), *Zfp708* maternal mutant (D) and *Zfp708* maternal-zygotic mutant (E) blastocysts, respectively.

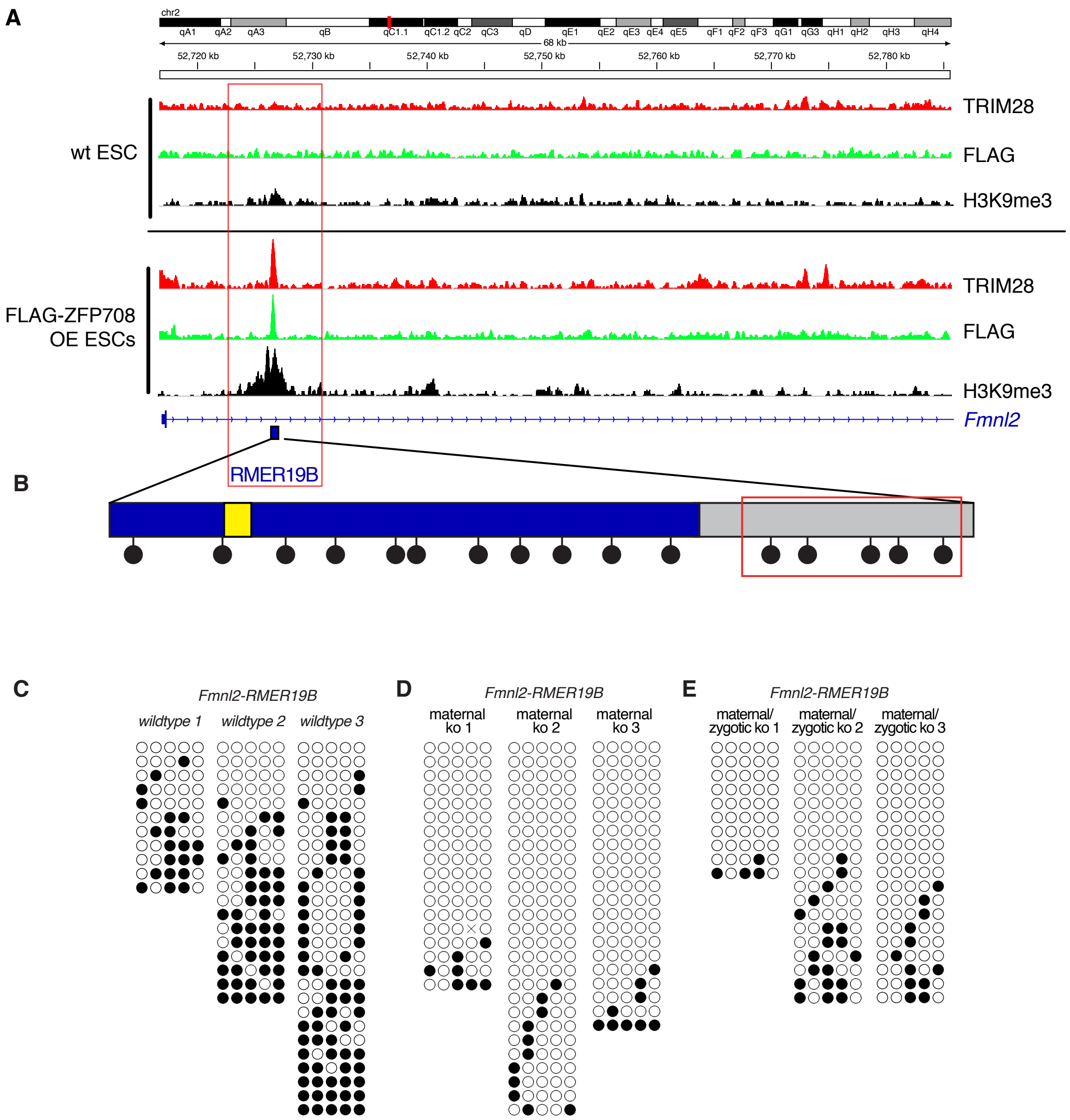
A
chr2
68 kb
52,720 kb
52,730 kb
52,740 kb
52,750 kb
52,760 kb
52,770 kb
52,780 kb
wt ESC
TRIM28
FLAG
H3K9me3
FLAG-ZFP708
OE ESCs
TRIM28
FLAG
H3K9me3
Fmnl2
RMER19B
B
C
Fmnl2-RMER19B
wildtype 1
wildtype 2
wildtype 3
D
Fmnl2-RMER19B
maternal ko 1
maternal ko 2
maternal ko 3
E
Fmnl2-RMER19B
maternal/ zygotic ko 1
maternal/ zygotic ko 2
maternal/ zygotic ko 3

Figure S10. DNA methylation analysis at the *Fmnl2-RMER19B* locus (A) Genome browser view of ZFP708, TRIM28 and H3K9me3 enrichment in wildtype and FLAG-ZFP708 OE ESCs at the *Fmnl2* gene locus. The RMER19B integration in the first intron of *Fmnl2* is highlighted (red box). (B) Schematic representation of the *Fmnl2-RMER19B*. The binding site is labelled in yellow, CpG sites are indicated as lollipops, the analyzed CpGs in the RMER19B-adjacent region (grey) are boxed in red. (C-E) DNA methylation state at the indicated CpGs in three independently derived ESCs lines from wildtype (C), *Zfp708* maternal mutant (D) and *Zfp708* maternal-zygotic mutant (E) blastocysts, respectively.

Development • Supplementary information

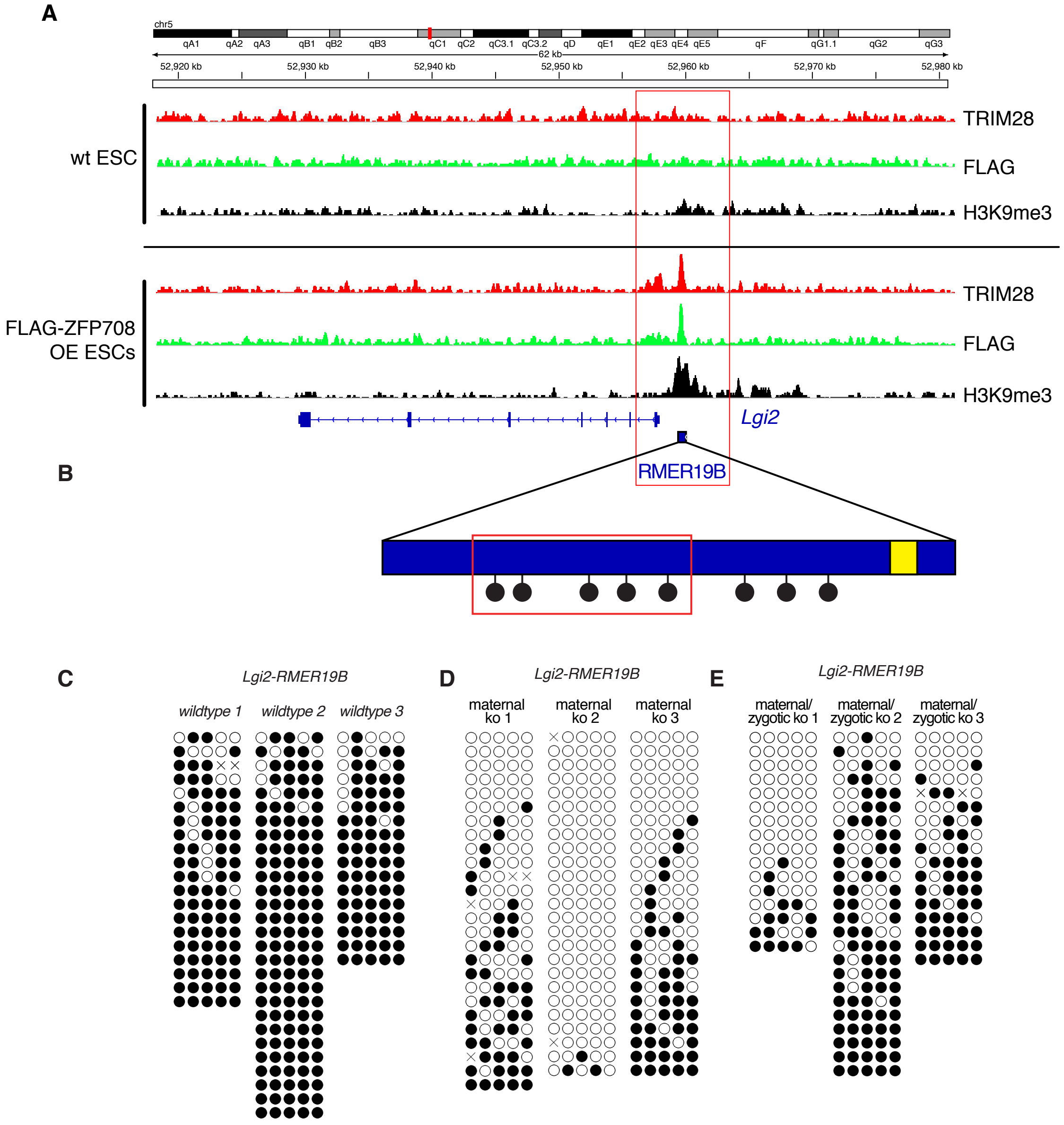

Development • Supplementary information

Figure S11. DNA methylation analysis at the *Lgi2-RMER19B* locus

(A) Genome browser view of ZFP708, TRIM28 and H3K9me3 enrichment in wildtype and FLAG-ZFP708 OE ESCs at the *Lgi2* gene locus. The RMER19B integration in the promoter region of *Lgi2* is highlighted (red box). (B) Schematic representation of the *Lgi2-RMER19B*. The binding site is labelled in yellow, CpG sites are indicated as lollipops, the analyzed CpGs are boxed in red. (C-E) DNA methylation state at the indicated CpGs in three independently derived ESCs lines from wildtype (C), *Zfp708* maternal mutant (D) and *Zfp708* maternal-zygotic mutant (E) blastocysts, respectively.

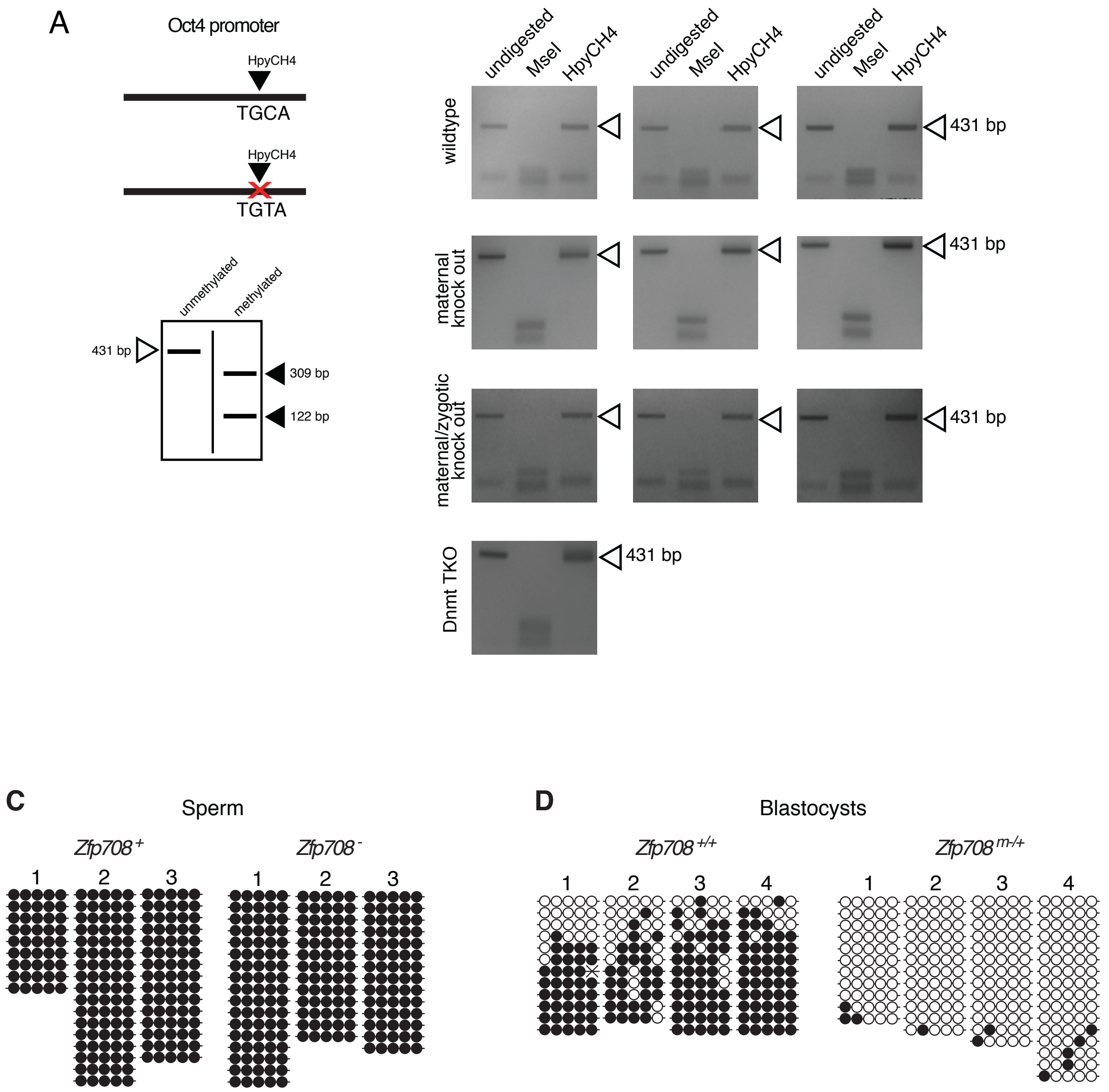

Development • Supplementary information

Figure S12. DNA methylation analysis

(A) COBRA analysis of the *Oct4* promoter region in three wildtype, ZFP708 maternal and ZFP708 maternal/zygotic knock out mESCs. If methylated COBRA produces two DNA bands (309 and 122 bp), if unmethylated COBRA produces one DNA band (431 bp). All mESC lines examined show full hypomethylation at the *Oct4* promoter. DNMT TKO mESCs serve as hypomethylation control. (C) DNA methylation analysis of the *Uck2*-RMER19B locus in three independent control and mutant derived sperm samples, respectively. (D) DNA methylation analysis of the *Uck2*-RMER19B locus in four pooled litters of control and maternal knock out ZFP708 E3.5 blastocysts, respectively.

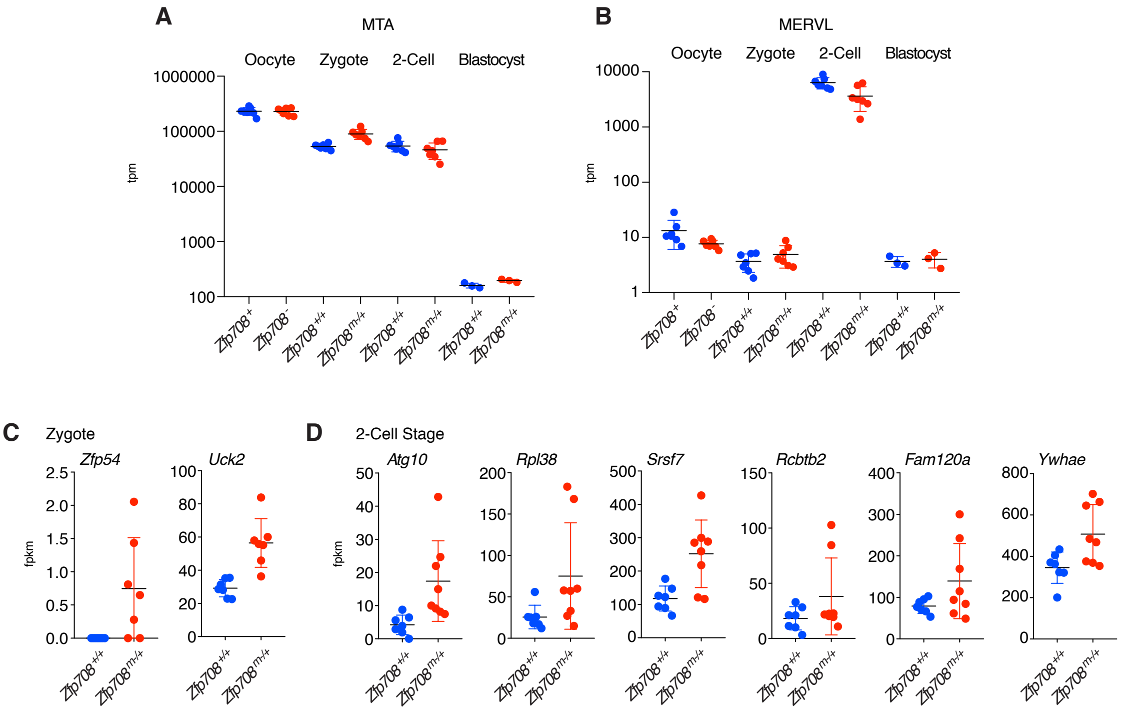

Figure S13. Transcriptional effects

(A-B) RNA-seq expression analysis of MTA (A) and MERVL (B) EREs during preimplantation development in ZFP708 mutant and control embryos. MTA and MERVL elements are analyzed as controls as they show very defined expression domains in preimplantation embryos. (C) Two ZFP708-bound RMER19B adjacent genes (within 50kB of peak) show significant upregulation in zygotes. (D) Six ZFP708-bound RMER19B adjacent genes (within 50kB of peak) show significant upregulation in 2-cell stage embryos. (tpm, transcripts per million mapped reads; fpkm, fragments per kilobase of transcript per million mapped reads)

Development • Supplementary information

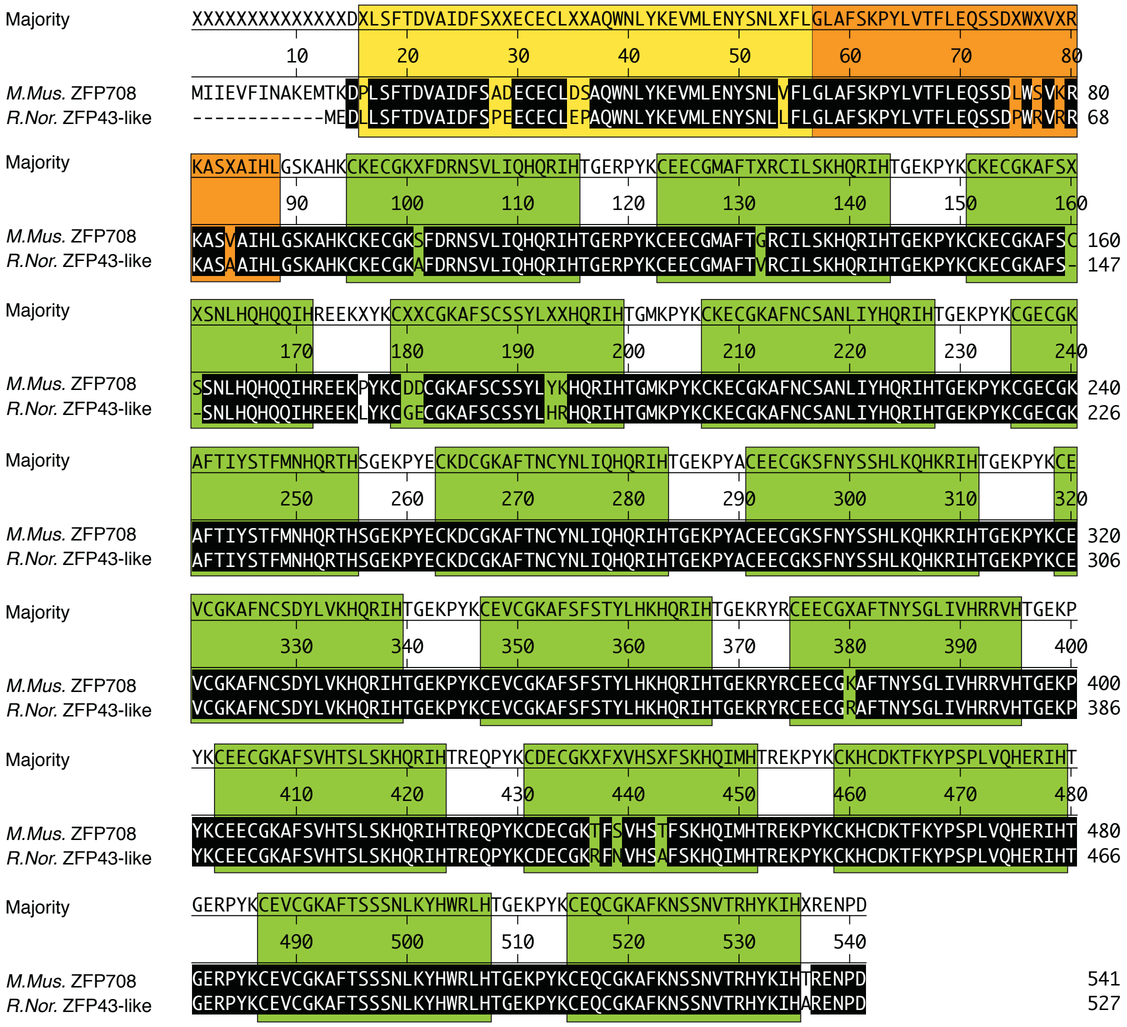

Figure S14. Mouse Rat conservation amino acid sequence alignment. Alignment of *M. musculus* ZFP708 and closest *R. norvegicus* orthologue (ZFP43-like) protein sequence. The typical N-terminal KRAB-domain consisting of a KRAB-A and a KRAB-B box are labelled in yellow and orange, respectively. The 16 classical C2H2-Zinc fingers are labelled in green. Matching amino acid residues are boxed in black.

Click here to Download Tables S1 - S15
